# Impact of a large-scale interactive and immersive science pop-up shop about infection and hygiene on visitors and volunteers

**DOI:** 10.3389/fpubh.2026.1812530

**Published:** 2026-04-23

**Authors:** Jonathan M. Tyrrell, Abdulsalam Abdulgafar, Pouya Sadeghzadeh Rahimi, Georgina B. Sparling, Friday K. A. Cox, Lila-Blythe Maros, Jeff Lewis, Naomi Stanton, Sarah Hatch, Carl Smith, Simone M. Cuff, Rebecca J. Aicheler, Matthias Eberl

**Affiliations:** 1 Institute of Life Science, Swansea University Medical School, Swansea, United Kingdom; 2 Division of Infection and Immunity, School of Medicine, Cardiff University, Cardiff, United Kingdom; 3 School of Biosciences, Cardiff University, Cardiff, United Kingdom; 4 Cardiff School of Sport and Health Sciences, Cardiff Metropolitan University, Cardiff, United Kingdom; 5 Centre for Medical Education, School of Medicine, Cardiff University, Cardiff, United Kingdom; 6 Public Involvement and Engagement Team, School of Medicine, Cardiff University, Cardiff, United Kingdom; 7 The Research Service, Cardiff University, Cardiff, United Kingdom; 8 Research Integrity, Governance and Ethics, Cardiff University, Cardiff, United Kingdom; 9 Systems Immunity Research Institute, Cardiff University, Cardiff, United Kingdom

**Keywords:** antimicrobial resistance, civic mission, education, health, hygiene, infection, public engagement, school outreach

## Abstract

Infectious diseases pose persistent and evolving challenges to global health, exacerbated by rising antimicrobial resistance (AMR) and vaccine hesitancy. The global public response to the COVID-19 pandemic underscored that scientific knowledge alone cannot drive behavioural change without trust, dialogue and meaningful social participation. Effective science communication and public engagement therefore require interactive, community-centred approaches that address social and cultural barriers and foster shared understanding and collective responsibility. This study describes the development and evaluation of a free large-scale pop-up shop designed to improve public health literacy. Located in one of the UK’s busiest shopping centres, the event was co-created and co-delivered by staff and students across four universities and combined playful, sensory, immersive learning with robust science through hands-on activities, exhibitions and live presentations. Incentives such as a sticker-based reward system and a treasure hunt encouraged sustained engagement. Over a 28-day period, 4,955 people attended, including organised school groups. Mixed-methods evaluation revealed positive shifts in public knowledge and attitudes, alongside an increased appreciation of the value of public engagement among those delivering the event. Embedding science within a public space proved effective in engaging diverse audiences, stimulating conversations around health and disease, and breaking down perceived barriers between scientists and the public.

## 1 Introduction

The global challenges posed by infectious diseases, fuelled by the concerning rise in antimicrobial resistance (AMR) and vaccine hesitancy, underscore the need for effective and sustained public engagement with science (1). Despite advances in biomedical research, clinical practice and public health measurements, a gap persists between scientific knowledge and public understanding, manifested through misconceptions about antibiotics, vaccine misinformation and inconsistent hygiene practices (1–3). The COVID-19 pandemic reaffirmed that knowledge alone is not sufficient to effect behavioural change, and that the effectiveness of infection prevention and control strategies depends not only on technology and policy but also on public trust, active participation in decision making and empowering individuals to make informed choices (1, 4).

Infection prevention and hygiene remain among the most effective health interventions—from handwashing to infection control within healthcare and community settings, adherence to hygiene practices, vaccination schedules and an understanding of the principles of antibiotics stewardship substantially reduces disease transmission (5). However, sustaining these behaviours requires motivation and supportive social norms. Public engagement is central to bridging this behavioural gap where interactive communication, visual storytelling and participatory experiences can render infection biology tangible and relatable (6–12). Initiatives enabling individuals to visualise bacterial growth, contamination or the importance of handwashing have the potential to have lasting impacts on perceptions and attitudes (13–15). Engaging stakeholders in immunology and microbiology thus extends beyond mere science dissemination; it is a two-way process that addresses social and cultural barriers to health literacy and empowers individuals and communities to make informed health decisions (16).

AMR represents one of the most critical global health threats of the 21st century. The often inappropriate use of antibiotics in communities, veterinary medicine and agriculture has accelerated the emergence of resistant pathogens, undermining the efficacy of essential treatments and invoking the potential ‘end of modern medicine’ (17–19). Despite growing scientific and policy attention, public understanding of AMR remains limited, and addressing AMR effectively requires communication strategies that move beyond the traditional top-down model (14, 20). Participatory engagement through citizen science projects, exhibitions and community workshops can help people see AMR as a shared social responsibility rather than an abstract scientific problem (12, 14, 21–23).

Of similar, or arguably even greater, relevance for containing and controlling infectious diseases, compared to antibiotics, are safe and effective vaccines (24). Vaccination is one of the most astonishing achievements for global health, yet its success is to a large extent contingent on public confidence. In fact, vaccine hesitancy, often driven by cultural beliefs, circumstantial misinformation, deliberate disinformation and distrust in institutions, poses a major barrier to immunisation against preventable diseases (1, 25). The COVID-19 pandemic demonstrated the life-saving efficacy of vaccines, and at the same time exposed the fragility of public trust. Although scientific consensus affirms vaccine safety and effectiveness, misinformation and disinformation spread rapidly via digital media, influencing perceptions and behaviours on a global scale (1). Countering those requires engagement that fosters transparency and reciprocity rather than one-way information delivery. Encouraging public dialogue about vaccine development, testing and monitoring is therefore critical to breaking down perceived barriers, building trust and strengthening social commitment to vaccination (4, 25).

Historically, science communication has been dominated by the ‘deficit model’, which positions the public as uninformed and scientists as authoritative knowledge providers. It assumes that increased knowledge automatically leads to behavioural change—a view now recognised as overly simplistic (26, 27). Modern science communication recognises the importance of dialogue, trust and contextual understanding through the lens of personal values, experiences and cultural identities. Engagement strategies that acknowledge these dimensions are more effective in fostering behavioural change and public trust (28). An emerging and innovative approach within this paradigm is the concept of ‘taking science to the city’, creating interactive, community-based environments in public spaces that democratise access to scientific dialogue (22). Pop-up science shops exemplify this approach, combining education, art and conversation to make complex topics accessible and engage audiences who might not typically seek out scientific experiences, thereby reaching diverse demographic groups beyond traditional academic or museum settings (29).

We here describe the creation of a large-scale pop-up shop to increase public awareness and understanding of topics related to infection, hygiene, AMR and vaccination. We combined our previously validated approach offering a range of interactive games and activities (14) with additional novel content such as a microbial ‘treasure hunt’ developed earlier (15), a newly conceived immersive ‘horror show’, a live stage for presentations and discussions, an art exhibition, complementary online information, and the possibility to invite and host local pupils and their teachers on educational school trips. We describe the delivery of inspiring content and activities, the underlying ambitions and objectives, and a full evaluation of the learning outcomes and impact of the event both on visitors and on students and staff volunteering at the event. We believe our approach and our findings are relevant to anyone interested in engaging diverse demographics in research, innovation and technology, and transferrable to other areas of knowledge and other communities and countries.

## 2 Methods

An exact description of how the event was designed and delivered can be found in the Supplementary information; detailed information and a comprehensive photo album are also accessible on the pop-up shop website.^1^ Evaluation of the event used a pragmatic mixed-methods approach incorporating quantitative measurements and qualitative feedback, alongside longitudinal follow-up data. Triangulation followed a convergent (parallel) design for defining the immediate impact on visitors and volunteers; a longitudinal triangulation design served to gauge retention of knowledge and reflections on lasting impact in a subgroup of visitors.

General metrics such as the number of visitors and helpers, sticker challenges and treasure hunts completed, body swabs grown and prizes earned were captured every day. Daily enumeration of answers to benchmarking questions was used to quantify pre-existing knowledge and learnings. Visitors were invited to provide feedback on a printed questionnaire at the end of their pop-up shop visit or alternatively complete an online version of it. Visitors who left their contact details for future follow-ups were contacted 4.5 months after the closure of the pop-up shop to provide their views on the long-term impact of the event, using a Microsoft Forms survey. Anecdotal feedback was collected via post-it notes left on the pop-up shop’s bacterial thought tree as well as via verbal communication, social media interactions and emails received during or after the event. Helpers were encouraged to provide feedback on their experience of volunteering at the event, using a Microsoft Forms survey. Web traffic on the Superbugs internet domain^2^ was analysed using Squarespace and Google Console. Historical weather data were extracted from public records provided by Meteostat.^3^ Distances between children’s schools and the venue were calculated as shortest driving distances using Google Maps. Graphs were created using Microsoft Excel and GraphPad Prism; statistical differences were assessed using GraphPad Prism using the tests described in the corresponding figure legends.

## 3 Results

### 3.1 Concept

The main objective of this pop-up shop was to increase public awareness and understanding of hygiene, infection and immunity—promoting reflection on health, responsibility and collective wellbeing, and breaking down perceived barriers between scientific experts and members of the public. Secondary objectives included the evaluation of the impact of an interactive pop-up shop on a cross-section of society in a busy shopping centre, and likewise the impact on volunteers helping in the creation and delivery of the event. The project allowed us to establish partnerships with primary and secondary schools and local businesses, and fostered closer cooperation across higher education institutions in the region. We also successfully created training opportunities for undergraduate students, early career researchers and educators to further their communication and team working skills, and used the opportunity to identify potential barriers and facilitators for their participation in public engagement activities.

### 3.2 Content and delivery

The Superbugs pop-up shop was hosted in a vacant 891 m^2^ retail unit at St David’s Dewi Sant shopping centre in Cardiff, one of the UK’s busiest regional shopping centres with an annual footfall of around 30 million visitors (Supplementary Figure 1). In total, Superbugs occupied this retail unit for 10 weeks, including 4 weeks of setting up content and activities, 4 weeks of in person delivery, and 2 weeks of dismantling the shop. A previous, smaller pop-up shop held in 2019 just a few retail units away from the current space had already established the core principles of the approach, receiving very positive feedback and generating clear learnings (14). Notably, visitor recommendations for future iterations had consistently called for *more*—a bigger pop-up, richer content and greater interactivity; in fact, re-analysis of those data from 2019 showed that *more* had been the most frequently used word in the feedback (Supplementary Figure 2). We took this enthusiasm as encouragement to retain the most successful elements of the original shop layout while expanding it considerably in scope, scale and duration.

The 2025 pop-up combined scientific information with hands-on, family-friendly activities. Large wall-mounted infoboards covered key scientific aspects of the microbial world (Supplementary Figure 3), alongside a further 100 explanatory posters and signs. Seven main interactive activity stations formed the core of the experience (Supplementary Figure 4). These included growing microbes from body or environmental swabs, a hand-washing demonstration under UV light, microscope observations of different micro-organisms, a virtual reality ‘Space Lab’ simulating a biomedical laboratory, a ball pit explaining how the immune system recognises pathogens, a tin can alley game showing how AMR impairs the ability to treat infections, and a ring toss illustrating AMR gene transfers between bacteria. To encourage participation, visitors collected stickers at each station, earning small Superbugs-branded prizes for completing the challenge (Supplementary Figure 5); the educational content was reinforced through ‘Antibiotic Resistance Champion’ certificates promoting good antibiotic stewardship, a concept successfully used before (14). A recently developed treasure hunt (15) further motivated exploration, asking visitors to find hidden ‘nasty germs’ throughout the shop while learning about different microbial species and the diseases they cause (Supplementary Figure 6).

To engage a demographic different from young children, an immersive ‘horror show’ was housed in 11 converted fitting rooms of the former fashion store, where each cubicle focused on a globally important disease or theme, including AMR, vaccine-preventable childhood infections, malaria, parasitic worms, plague, cholera, typhoid, leprosy, COVID-19 and wound infections (Supplementary Figure 7). Displays combined dramatic presentation with accurate scientific explanations, highlighting both historical perspectives and modern medical advances. Additional features (Supplementary Figure 8) of the pop-up shop included a live stage, a school art exhibition (Supplementary Figure 9), an arts and crafts area (Supplementary Figure 10), a reading corner with popular science books and information (Supplementary Table 1) and numerous displays. Together, these pop-up shop elements went considerably beyond the previous event (14), and formed an inclusive, engaging and memorable public science experience designed to raise awareness of microbes, infectious diseases and the importance of antimicrobial stewardship and vaccination, while also inspiring a general interest in science, healthcare and biomedical careers. All written content was provided bilingually in English and Welsh throughout the event and complemented by extensive educational materials available online in both languages (30), to be inclusive of local cultural values that help ensure that Welsh and English are treated equally in public life in Wales.

### 3.3 Basic metrics

**Figure 1:**
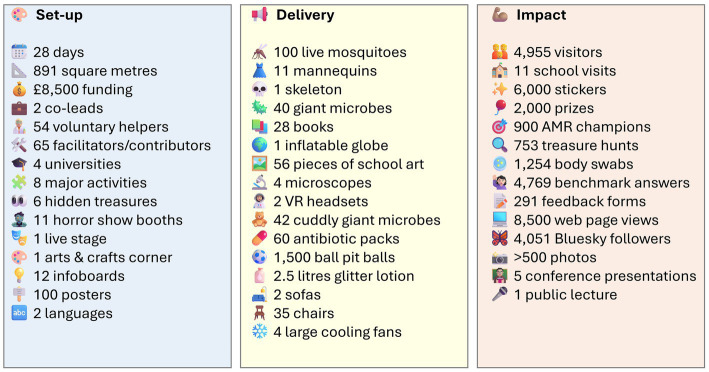
Overall metrics for the set-up, delivery and impact of the pop-up shop event.

The event operated daily from 9:30 to 16:30 from Monday 7 July 2025 until Sunday 3 August 2025. This period spanned the last 2 weeks of the Welsh school summer term and the first 2 weeks of the school summer holidays, including weekends. Over these 4 weeks, the pop-up shop attracted 4,955 visitors. Of these, 292 were pupils and teachers from seven English-medium schools and one Welsh-medium school who attended 11 pre-booked school visits (Figure 1). Between 12 July and 3 August 2025, 900 visitors (18.2%, daily range 11.5–36.8%) completed the sticker challenge, 753 (15.2%, daily range 7.4–28.7%) completed the treasure hunt; for technical reasons, no such data are available for the first 5 days of the event. With 151 pupils and teachers visiting between 7 July and 11 July 2025, and given that one of the sticker challenge activities involving the growing of body and environmental swabs used 1,254 agar plates (25.3% of all visitors, daily range 16.2–65.3%), actual engagement with the pop-up shop content was above that measured. Overall figures were comparable to our event in 2019, when 24.8% of all visitors completed a similar sticker challenge, and 33.0% participated in a similar ‘Grow your own microbe’ activity (Supplementary Figure 11A)—albeit in a more basic setting, with no treasure hunt or horror show distracting from the sticker challenge (14). Of note, the treasure hunt was marginally less popular than the sticker challenge (Supplementary Figure 11B), possibly because it targeted a younger audience whereas the sticker challenge was designed for all ages.

In addition to bringing science to the city and reaching large numbers of member of the public, an equally important aim of the pop-up shop was to train university staff and students in engaging effectively with the public. This included 54 volunteers from Cardiff University, Cardiff Metropolitan University, Swansea University, Bristol University and beyond, as well as in-kind contributions from an additional 65 facilitators, demonstrating the complexity and ambition of the event (Figure 1).

**Figure 2:**
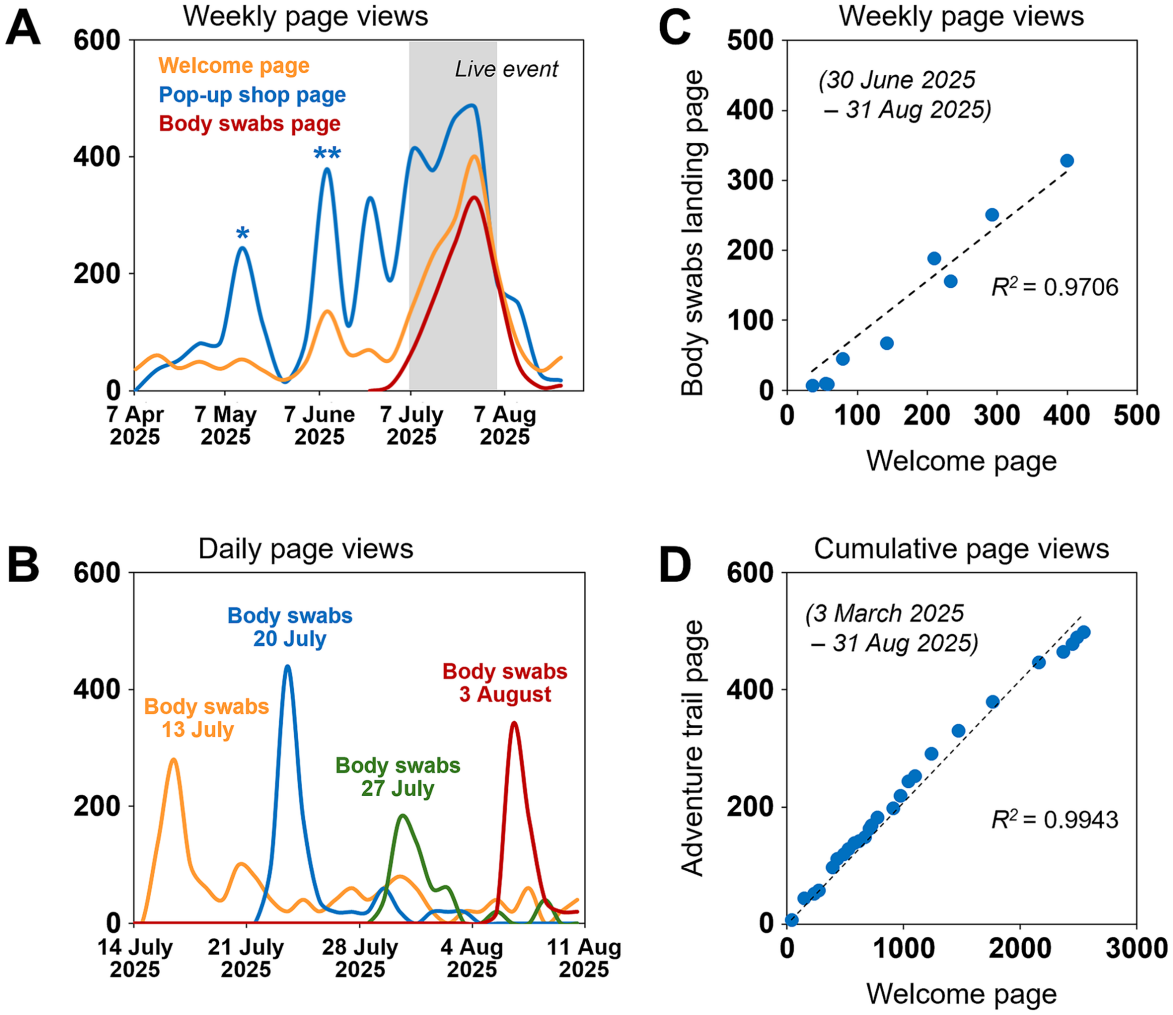
Web traffic on the Superbugs website. **(A)** Weekly page views on the Superbugs welcome page: www.superbugs.online (orange), the event main page: www.superbugs.online/pop-up-2025 (blue), and the landing page for the body swabs: www.superbugs.online/pop-up-2025/body-swabs (red)—shaded area, duration of the live event from 7 July to 3 August 2025, ^*^ time of Superbugs email campaign targeting teachers and schools across South Wales, ^**^ time of press release and social media campaign by St David’s Dewi Sant; **(B)** daily page views on the body swab results pages for swabs taken on 13 July (orange), 20 July (blue), 27 July (green) and 3 August 2025 (red); **(C)** correlation between weekly views on the welcome page and the body swabs landing page, for the period from 30 June to 31 August 2025; **(D)** correlation between cumulative views on the welcome page and the Adventure Trail start page: www.superbugs.online/activities, the main educational feature of the Superbugs website, for the period from 3 March to 31 August 2025.

Web traffic to the Superbugs website showed a striking peak at the time of the event, demonstrating that the event directly forwarded people to the online content. This was to a large extent due to the event-specific page that had been developed and promoted over the preceding 3 months, with sharp peaks in traffic coinciding with major email campaigns before the event (Figure 2A). Traffic to the landing page for the body swab results^4^ matched the pop-up shop activities, with daily page views suggesting that visitors consistently accessed the swab results 2–4 days after their visits (Figure 2B). Page views for the body swabs landing page correlated with views of the Superbugs welcome page over the duration of the event, confirming that visitors accessed the swab results via the welcome page, as instructed by the pop-up shop helpers (Figure 2C); as reference, page views for the ‘adventure trail’—the Superbugs website’s main educational content—similarly correlated with long-term traffic on the welcome page (Figure 2D). Overall, these data demonstrate the successful combination of in-person activities with complementary online content.

Closer examination of the daily fluctuation of visitor numbers and activities in the pop-up shop for the public period from 19 July until 3 August 2025 suggested a correlation with the weather. Overall, we saw a trend towards more visitors and more completed sticker challenges and treasure hunts on rainy days compared to dry days (Supplementary Figure 12A). This difference was particularly apparent for the number of completed treasure hunts (Supplementary Figure 12B)—an activity that predominantly engaged small children and thus was deemed good indicator for visits by young families. No such trend was apparent for the relationship between visitor numbers and outside temperatures (not shown), suggesting that rainfall was the more obvious factor affecting footfall.

When measuring impact of social media advertising on footfall, it was of minor effectiveness in driving traffic to the pop-up shop. The event was promoted online in advance and while it was ongoing, by the Superbugs team itself as well as by Cardiff University, St David’s Dewi Sant, Visit Cardiff, Cardiff Passport to the City, ITV Wales, visiting schools and several pop-up shop helpers across Facebook, X/Twitter, Bluesky, Instagram, TikTok and LinkedIn (Supplementary Table 2). The vast majority of social media posts had only a very modest impact with 5–20 likes, with Instagram anecdotally achieving the highest number of interactions with up to 358 likes—still low compared to the total number of visitors.

### 3.4 Quantitative impact evaluation of learning outcomes

A benchmarking exercise served to assess learnings, by asking visitors the exact same questions upon entering the pop-up shop, and again when leaving (Supplementary Figure 13). This approach allowed us to gauge the pre-existing knowledge of what type of infections can be treated with antibiotics, whether visitors understood what the term ‘antimicrobial resistance’ means, and what they knew about how vaccines work. By asking visitors the same questions again after having explored our activities and displays we tested whether their answers had changed and thus, whether the experience of our event had improved their understanding of antibiotics, AMR and vaccines. Overall, more than 1,000 people (i.e., roughly 1 in 5 visitors) answered the benchmarking questions before engaging with the pop-up shop and over 500 answered the questions upon leaving the shop.

**Figure 3:**
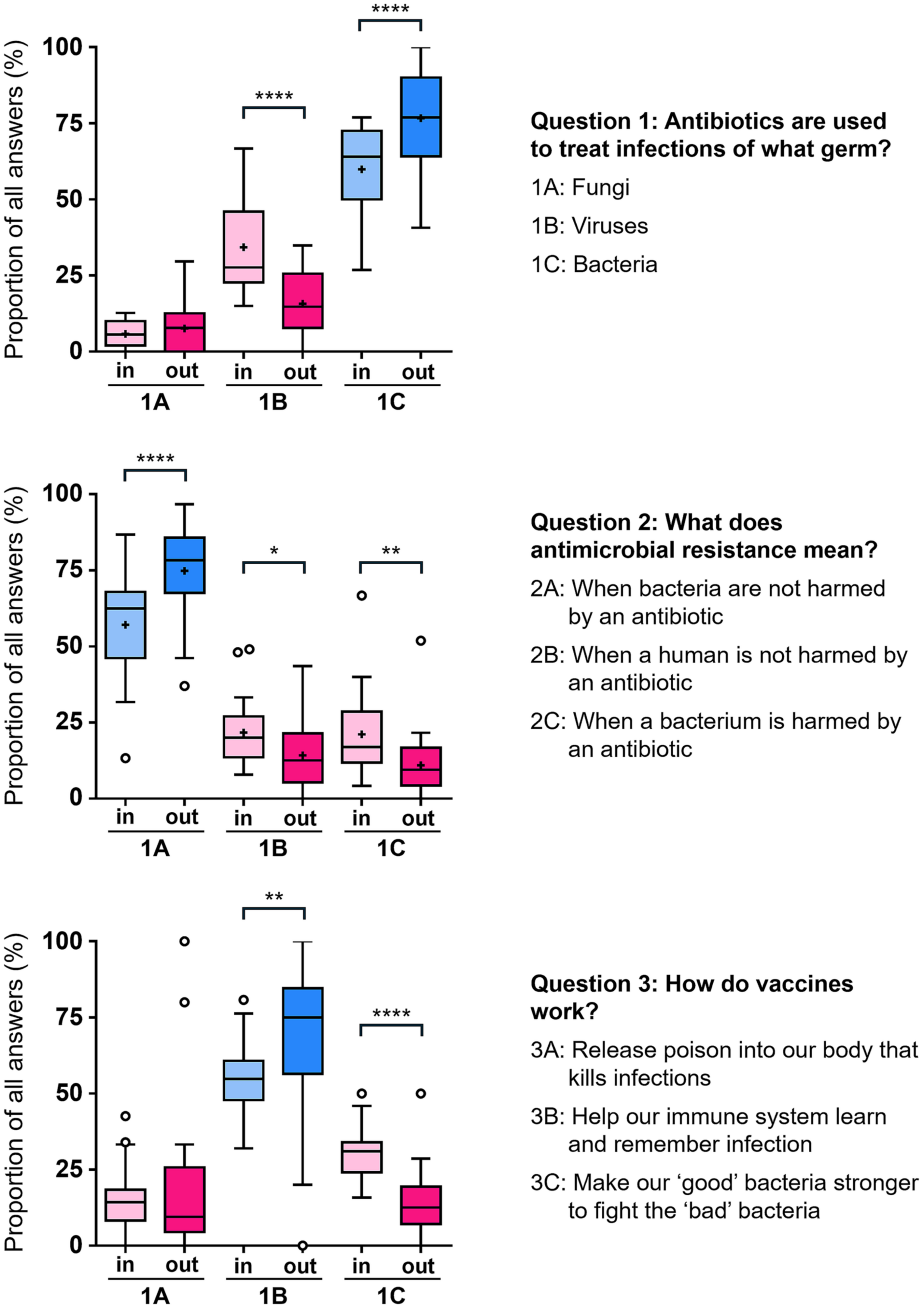
Assessment of learning outcomes. Each day, visitors were asked the three questions shown at the beginning (in; *n* = 1,080, 1,015, and 1,058, respectively) and end of their visit (out; *n* = 563, 524, and 529, respectively), over 27 days. Day 1 of the pop-up shop was excluded from the analysis as no exit data had been collected that day. Correct answers are shown in blue, wrong ones in pink. Tukey whisker plots show upper and lower quartiles as boxes above and below the median line, and whiskers representing the 1.5 interquartile range. Outliers are depicted as individual data points, the mean by the plus symbol. Statistical significance was assessed using RM one-way ANOVA with Greenhouse–Geisser correction and Holm-Sidak’s test for multiple comparisons: *p < 0.05; **p < 0.01; ***p < 0.001; ****p < 0.0001.

On entry to the pop-up shop, up to half of all visitors were not aware that antibiotics are used against bacteria not viruses or fungi, that AMR means that bacteria are not harmed by antibiotics, and that vaccines help our immune system learn and remember infections (Figure 3). The bottom range of correct pre-visit answers on some days was as a low as 1 in 4 visitors. Percentages of correct answers when exiting the shop were significantly higher than this baseline, with an average knowledge improvement by 17, 18 and 14%, respectively, for the three questions (Figure 3). In addition to the significant increases in correct answers as a result of exploring the pop-up shop, there was a parallel drop in some false answers, in particular with regard to the number of visitors who had originally, and wrongly, thought that antibiotics are used to treat viral infections. Encouragingly, this positive learning experience of the total visitor cohort was replicated when limiting the impact analysis to seven fully evaluated school visits, with similar trends in learning outcomes (Supplementary Figure 14).

School visits also gave an opportunity to assess learnings by conducting a quiz in groups of 2–4 pupils at the end of the tour, with a prize for the highest scoring team. Questions related to aspects of activities and displays, many of which could only be answered correctly upon engaging with the corresponding content. Perhaps unexpectedly, relatively simple questions were answered more easily (e.g., “What do we use to look at micro-organisms more closely?”—Microscope, 96.7%) than questions relating to scientific knowledge (e.g., “What type of bacteria looks pink under the microscope?”—Gram-negative, 71.1%; Supplementary Figure 15). Yet, each question was consistently answered correctly by at least two thirds of the teams. Out of 90 teams in total, 27 (30.0%) got all eight questions right, a further 26 (28.9%) seven out of eight questions. These high scores were all the more encouraging as visits came from both primary and secondary schools, demonstrating learning across all ages.

### 3.5 Qualitative evaluation through visitor questionnaires

**Figure 4:**
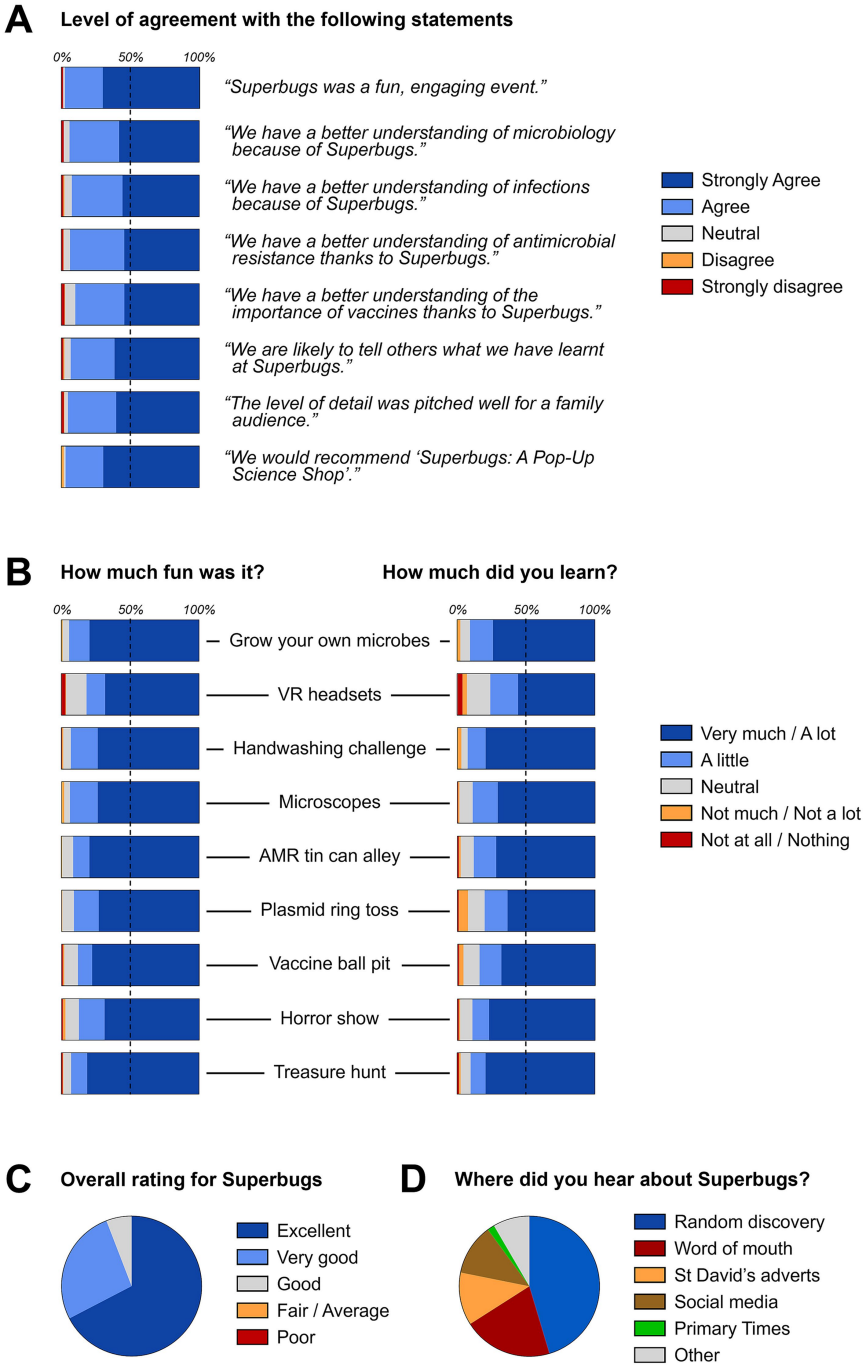
Quantitative feedback by pop-up shop visitors. **(A)** General experience; **(B)** Ratings for individual activities; **(C)** Overall rating; **(D)** Original source of information about the event. Information collated from a total of 244 completed post-event questionnaires.

Qualitative feedback was captured via questionnaires that could be completed in person at the end of the regular pop-up shop visit (30.2% of all responses) or in an online format (69.8%), depending on personal preferences. This feedback from a total of 244 respondents (corresponding to 5.2% of all 4,663 public visitors) was overwhelmingly positive. It should be noted that questionnaires were often completed on behalf of whole families or groups of visitors; the responses obtained therefore represented a much larger proportion of actual visits than the 5.2% return rate would imply. The vast majority of respondents confirmed that they felt they had a better understanding of the topics presented, that the level of detail was pitched well, and that they would tell others about it and recommend the event (Figure 4A). Typically, over 90% of respondents agreed or strongly agreed with those sentiments, and merely 2.5% or fewer disagreed or strongly disagreed. Similarly, more than 3 out of 4 respondents stated that the individual activities had been fun, and that they had learnt something (Figure 4B).

It is notable that the activities that were rated as particularly ‘fun’ activities as judged by positive feedback—the ‘Grow your own microbes’ (94.5%) and microscopes (94.1%) stations, the handwashing challenge (93.2%) and the treasure hunt (93.1%; Figure 4B)—were also the highest scoring stations with regard to learning: ‘Grow your own microbes’ (90.8%), microscopes (88.9%), handwashing challenge (92.8%) and treasure hunt (90.4%). The virtual reality headset station rated lower than the physical activities, with 81.9% positive feedback for being fun and a positive learning outcome for 76.1% of respondents. Overall feedback was highly positive, with 67.4% of respondents giving an excellent overall ranking, the remaining 32.6% giving a very good or good ranking. None of the respondents considered the event to having been of only fair/average or even poor quality (Figure 4C).

Free-text feedback from visitors allowed a more nuanced evaluation to discover which elements of the pop-up shop created the strongest impressions (Supplementary Table 3). The feedback highlighted the overall enjoyability and interactivity of the activities (66.2%) and the enthusiastic, helpful staff (18.0%), contributing to a positive educational experience (18.9%); good accessibility for a wide range of audiences was also highlighted (14.9%; Table 1). Feedback on potential improvements in the future endorsed the current format, with nearly 30% of respondents saying that there was no need to improve anything. Among those who offered suggestions, themes pointed to incremental enhancements rather than fundamental redesigns (Table 2). Suggestions for improvements included better advertising and scheduling (9.1%), more staff (8.4%) and ideas for additional formats such as interactive show performances, videos and audio explanations (7.8%).

**Table 1:** Qualitative feedback by visitors in response to the question “Please tell us what you liked most about Superbugs?”.

Theme and illustrative quotes
Interactivity and engagement (66.2%)
*“Very engaging - amazing displays - absolute fascinating.”*
*“Great free activity for kids, they loved the VR and also the coconut shy.”*
*“Loved the treasure hunt idea! The ball pit, hand washing station and the grow your own microbe were brill.”*
*“Great range all round, Microscopes and grow your own were great.”*
Educational value (18.9%)
*“We like that it was interactive, fun to learn!”*
*“I liked that it was fun and easy to understand how bacteria become resistant.”*
*“It was a fun and engaging way to introduce the topic to children. They were learning without realising!”*
Staff (18.0%)
*“The staff*!!*!! They were so engaging and animated.”*
*“Staff were lovely. My kids loved the experience. Well done all. Fantastic initiative.”*
*“The explanations from the volunteers helping – their enthusiasm and knowledge really b[r]ought the exhibitions to life and helped engage the children.”*
Accessibility (14.9%)
*“Made very complicated often adult topics accessible for our five year old.”*
*“Really interesting for children of all ages.”*
*“There was enough to keep younger children entertained even if they did not understand the msg in full.”*
*“It is amazing and informative for the kids and parents.”*
Value (4.5%)
*“How interactive it is, the staff were amazing and we learnt so much. All of this and it’s free!”*

The table shows major themes emerging from a total of 222 answers in the post-event questionnaires, alongside illustrative quotes.

**Table 2:** Qualitative feedback by visitors in response to the question “How could we improve Superbugs events in future?”.

Theme and illustrative quotes
No improvements needed (29.9%)
*“Nothing – it was great, my 5yo could not absorb everything, but he did learn and enjoyed it very much.”*
*“You did so well, I do not know what you could improve.”*
*“Keep it as it is.”*
*“It was perfect.”*
Comfort and environment (14.9%)
*“Outside your control, but it was too hot in the venue.”*
*“More VR headsets as both ran out of charge so could not do this in one go.”*
*“Light music in background.”*
*“Maybe have a gift shop as well.”*
*“Hand wash would be better if we were using soap and water rather than wipes.”*
Content and activities (13.0%)
*“Add more interactive games and hands-on activities.”*
*“Longer treasure hunt.”*
*“Even more activities!!!”*
Outreach and scheduling (9.1%)
*“Better advertising.”*
*“More advertisement and staying open for longer!”*
*“Adults-only evenings with wine and cheese.”*
*“Roll it out to other cities.”*
Staffing and facilitation (8.4%)
*“Have more people to talk to.”*
*“More staff although they tried very hard*: *-.”*
*“Get theatre students involved.”*
Interactivity and formats (7.8%)
*“Maybe an interactive show performance at certain times where you could sit and watch.”*
*“Include videos/presentations.”*
*“Audio explanation on every activity.”*
*“Put a map/list at the entrance with all the activities.”*

The table shows major themes emerging from a total of 154 answers in the post-event questionnaires, alongside illustrative quotes.

Anecdotal feedback reaffirmed the positive experience by visitors further, provided on post-it notes on the pop-up shop’s bacterial thought tree, verbally or by email throughout the event, as illustrated by two examples:

*“My children came home and drew lots of E. coli! We live in challenging times with a huge anti-science movement and it’s therefore vital that our children learn unbiased facts from experts as opposed to influencers and charlatans.” [email, day 8 of the event].*

*“We love you all so much! Thank you for this amazing experience!” [anonymous post-it note, day 25 of the event].*

Finally, having established the concept of an interactive pop-up shop on a previous occasion where we deliberately did not advertise the event and showed that a professionally designed venue is sufficient to attract passers-by (14), we asked visitors how they had found the present pop-up shop. Unexpectedly, out of 238 respondents, nearly half (45.4%) stated that it was a random discovery when walking past (Figure 4D), which was not much different from the 67.3% at our previous event (14). A further 20.6% heard about the event by word of mouth. Only 1 in 4 of responding visitors had come to the event because of adverts by the St David’s Dewi Sant team, social media posts or printed adverts in the Primary Times, a free magazine distributed to families of primary school-age children throughout Wales (Figure 4D). These findings are in accordance with the fact that most social media posts only reached low numbers of people (Supplementary Table 2). Together, these findings confirm the powerful draw of an attractive location in a busy shopping centre and the limited reach of our advertising campaigns.

### 3.6 Long-term impact on visitors

To gauge the longer-term impact of the event on visitors we created another survey that was distributed by email to 172 people, and via WhatsApp to a further 13, who had consented to be contacted in the original feedback questionnaire. People were contacted by the Superbugs team approximately 4.5 months after the closure of the pop-up shop. In total, this survey was completed by 21 former visitors; one further person replied by email: “We thought it was wonderful but no time for survey sorry” (together accounting for 11.9% of all contacted people, 9.0% of visitors completing the original questionnaire and 0.47% of all 4,663 public visitors). 95.2% of respondents who completed the full impact survey had visited the pop-up shop with children, typically one (47.6%) or two (33.3%), who were predominantly of primary school age (3–6 years, 51.4%, 7–10 years, 31.4%; Supplementary Table 4), together attending at least 23 different English and Welsh-medium primary and secondary schools. Respondents covered a wide area spanning South Wales and South-West England, showing the geographic reach of the event. The mean distance between the schools attended by the respondents’ children and the pop-up-shop location (calculated as driving distance using Google Maps) was 19.2 km, with a range of 1.1–124 km (Supplementary Table 4).

**Figure 5:**
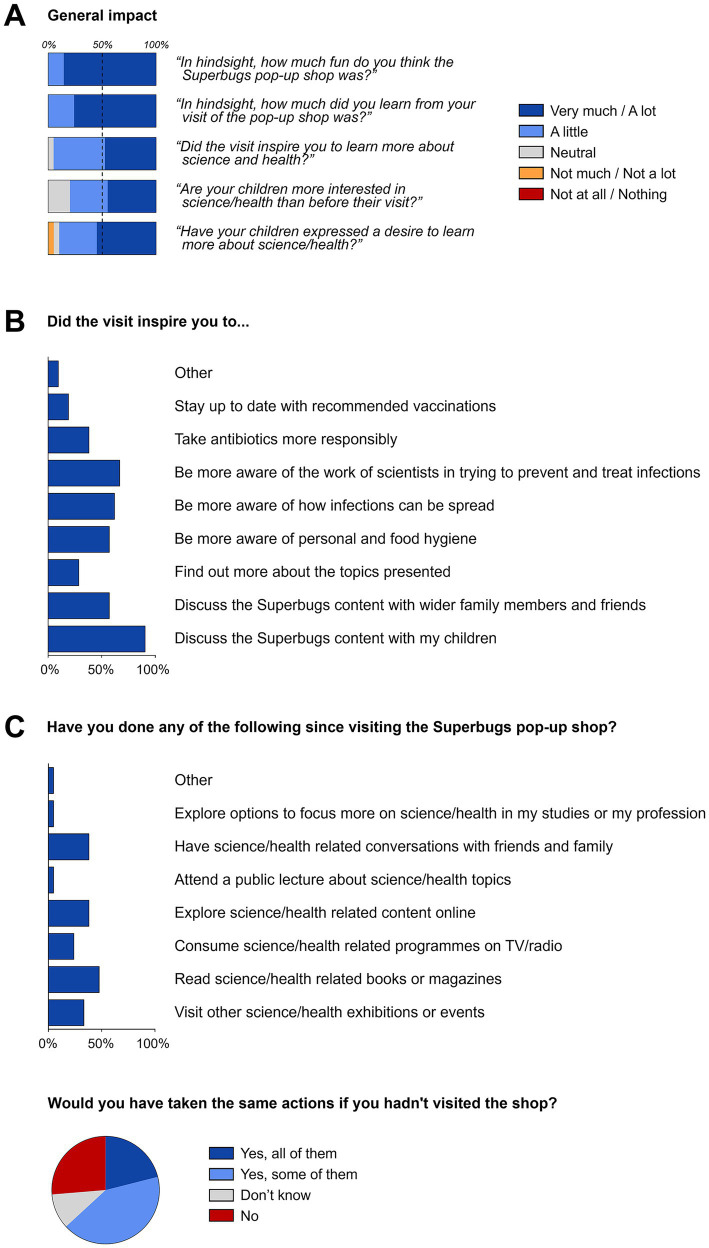
Long-term impact of the event on members of the public. **(A)** General impact; **(B)** change of perceptions and awareness (multiple choice); **(C)** direct actions taken (multiple choice). Information collated from 21 visitors providing feedback regarding their views 4.5 months after having visited the pop-up shop.

This subcohort of visitors not only thought that the pop-up shop had been fun and that they had learned something (100% each, not shown) but also that the visit had inspired them to learn more about science and health (95.2%; Figure 5A). 90.5% of survey respondents stated that after their visit they had discussed Superbugs content with their children (95.0% of respondents who actually visited the shop with children), 57.1% with their wider family and friends. More than half stated that they were now more aware of personal and food hygiene (57.1%), of how infections spread (61.9%), and of the work of scientists in trying to prevent and treat infections (66.7%; Figure 5B). Respondents also said that they had been inspired to take antibiotics more responsibly (38.1%) and to stay up to date with recommended vaccinations (19.0%; Figure 5B). Overall, respondents confirmed that they and/or their children had learned key messages (90.5%) and that some of the content had come up in conversation since their visit (95.2%; Supplementary Figure 16A), as clear demonstration of the long-term impact of the pop-up shop on visitors.

Many of the survey respondents confirmed that they had visited other science or health-related exhibitions or events after the pop-up shop event (33.3%), and explored science or health content in print (47.6%), on TV/radio (23.8%), online (38.1%) or by attending a public lecture (4.8%; Figure 5C). One respondent even confirmed that they had explored options to focus more on science or health topics in their studies or profession. Of note, only 19.0% of respondents taking these actions stated that they would have done the same if they had not visited the shop in the first place (Figure 5C). For almost 2 out of 3 survey respondents the shop had a lasting impact as they would have only taken some (38.1%) or none of these actions (23.8%) had they not visited the Superbugs event, demonstrating a clear shift in perceptions and interests (Figure 5C).

The children of the subcohort completing the long-term impact survey were now more interested (80.0%) and had expressed an interest in learning more about science and health (90.0%; Figure 5A). While the vast majority of respondents (90.0%) had not been aware of the Superbugs online resources, 42.9% had used it since visiting the pop-up shop; of those who had used it, 80.0% found the website useful for learning more about science and health while none of them found it not useful (Supplementary Figure 16B).

Finally, with regard to the pop-up shop approach, the majority of respondents stated that the location (90.5%) and the free nature of the event (95.2%) had been very important or somewhat important for their visit of the pop-up shop (Supplementary Figure 16C). Going forward, 95.2% would visit other pop-up shops about science, health or technology in the future (Supplementary Figure 16C), suggesting issues like “healthy eating,” “allergies,” “STEM for girls” [STEM = science, technology, engineering and mathematics], “space, ocean and pollution” and “the environment” as possible themes for future events, and listing free entry, child-friendliness, accessibility and fun as important criteria of scientific pop-up shops (Supplementary Table 5).

The following quotes from two respondents to the long-term impact survey illustrate the lasting impression the event had on children:

*“My daughter has talked about the event since and has mentioned the things that she learned in school to her teachers.”*

*“My son loved the day, he really enjoys sciencey things and still talks about the event. Whenever someone coughs he wonders ‘if they have the rhinovirus’.”*

### 3.7 Impact on helpers and volunteers

Given that the event not only attracted a large number of members of the public but was crucially dependent on a substantial number of voluntary helpers, at diverse career stages and from different higher education institutions, we aimed to evaluate the experience of volunteering at the event on this helper cohort. A total of 33 responses (61.1% of all helpers) to a post-event questionnaire were predominantly from undergraduate and postgraduate students (63.7%), but also included more senior research and teaching staff, professional service staff and A level pupils (Supplementary Table 6). The majority had a background in biomedical sciences (66.7%), others in medicine/healthcare or natural sciences. Importantly, most were quite inexperienced in delivering public engagement activities, with almost 3 in 4 having never participated in public engagement before, or only once a year. In the pop-up shop, helpers were predominantly involved in delivering activities to the general public (84.8%) and/or to school visits (51.5%), but some also contributed to planning and development of content or activities (27.3%), preparation of the location (21.2%) and evaluation (12.1%; Supplementary Table 6).

**Figure 6:**
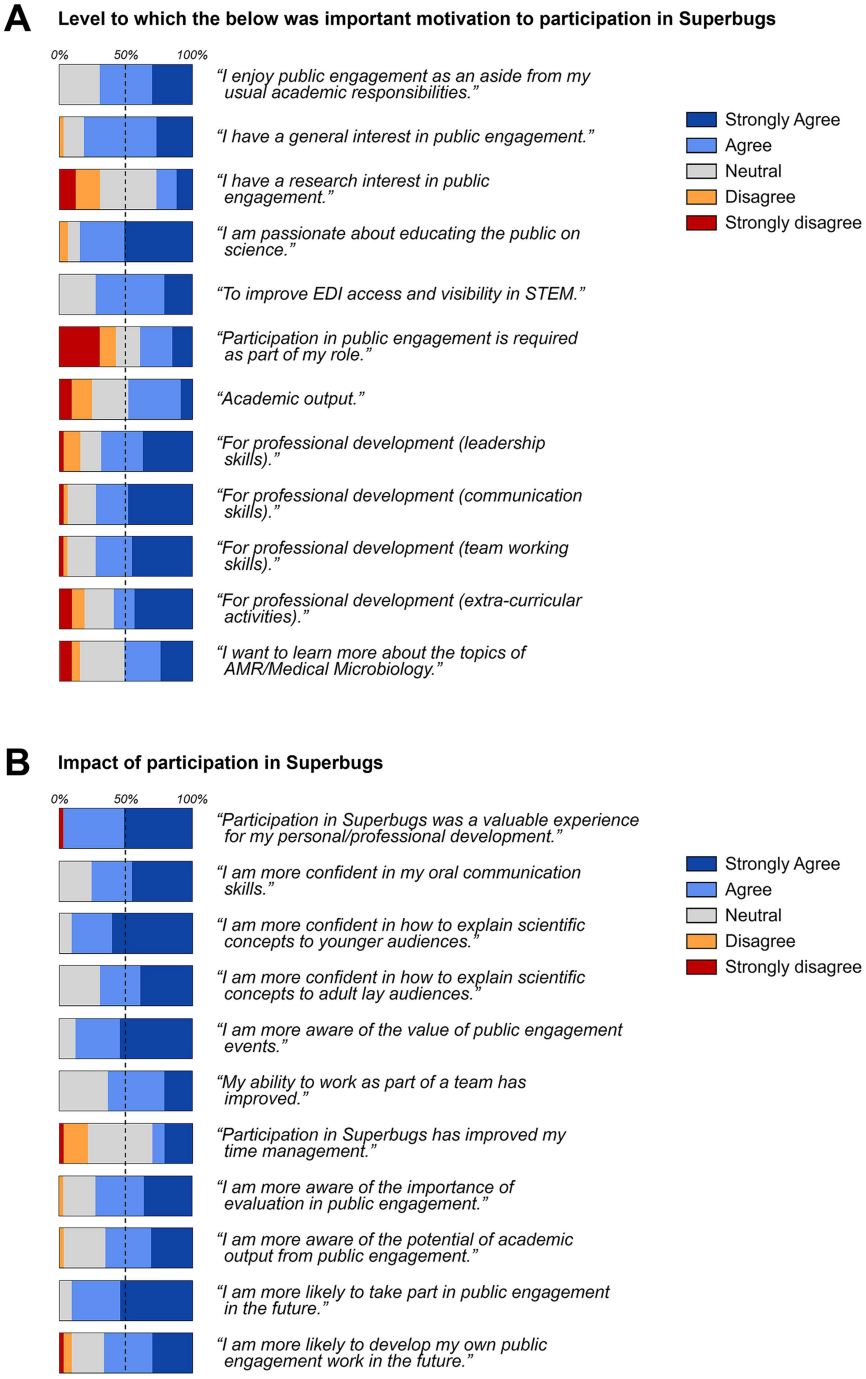
Quantitative feedback from helpers participating in the pop-up shop. **(A)** Motivation to participation; **(B)** impact of participation. Information collated from a total of 33 completed post-event questionnaires.

Many helpers responding to the post-event survey stated that they had mainly been motivated by their passion and interest in public engagement as an aside from their usual academic responsibilities (Figure 6A). Other motivators included opportunities for professional development with regard to communication, teamwork, leadership and extracurricular activities. Actual research interests and academic outputs in public engagement as well as a perceived requirement for participation in public engagement activities as part of their role ranked the lowest.

With regard to the impact of their participation, most helpers agreed or strongly agreed that the event had contributed to their personal and professional development and had improved their confidence in communicating science to younger and adult audiences and their team working skills (Figure 6B). Helpers also stated that they had become more aware of the value of public engagement, the importance of evaluation and the potential to produce academic outputs. The only aspect from the survey that the majority of helpers did not agree with was the positive impact of the event on their time management skills. Overall, 9 out of 10 helpers stated that after the pop-up shop they were more likely to participate in future public engagement events; no helper said that they were now less likely to participate. Moreover, 2 out of 3 stated that after the event they were more likely to develop their own public engagement activity in the future (Figure 6B).

Helpers had a very positive perception of the pop-up shop overall and largely agreed that the event had provided an engaging learning environment and a safe space for diverse audiences (Supplementary Figure 17A). With regard to their experience of volunteering at the event, most helpers agreed or strongly agreed that they had been given adequate information and induction before their participation (Supplementary Figure 17B). Reassuringly, all helpers had felt supported and valued by the team, and able to ask questions; none of the helpers disagreed. Despite the perceived lack of sufficient helpers, the workload had mainly been to the level expected (Supplementary Figure 17C).

**Figure 7:**
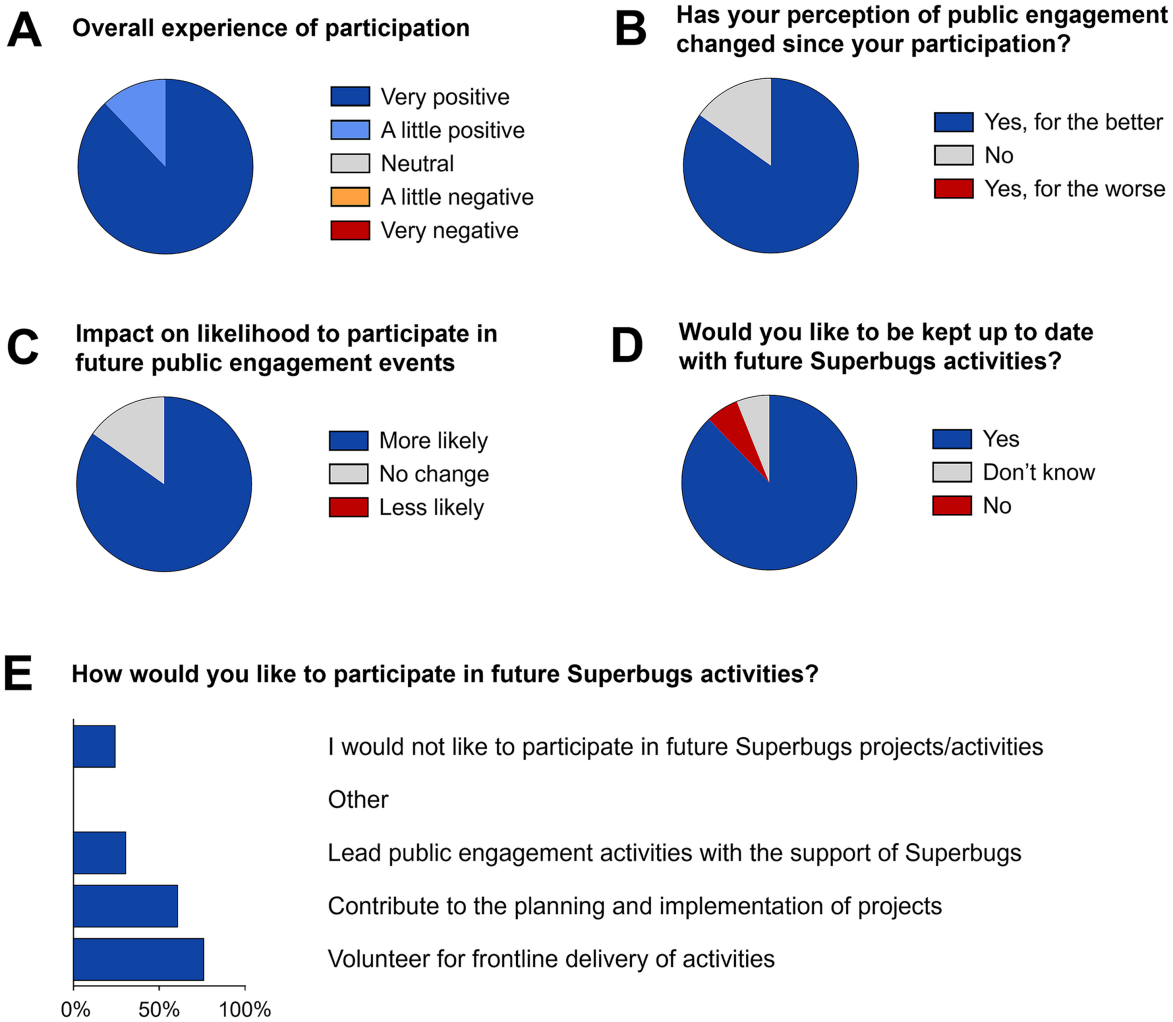
Impact on helpers participating in the pop-up shop. **(A)** Overall experience of participation; **(B)** change of perception of public engagement; **(C)** likelihood to participate in future of public engagement activities; **(D)** willingness to be kept up to date with future Superbugs activities; **(E)** type of potential participation in future Superbugs activities (multiple choice). Information collated from a total of 33 completed post-event questionnaires.

All Superbugs participants who rated their overall experience found it to be positive, with 87.9% rating it as ‘very positive’ (Figure 7A). Reassuringly, no helper rated their experience as neutral or negative, indicative of the positive learning and training environment for helpers. 84.8% of respondents stated that their perception of public engagement had changed for the better (Figure 7B), and that they were more likely to participate in future public engagement event (Figure 7C); no respondent said that their perception had changed for the worse or that they were less likely to participate. The lasting relationship we established with our helpers is illustrated by the fact that 87.9% would like to be kept up to date with future Superbugs activities (Figure 7D). Even more, 75.8% of all respondents would like to volunteer again for frontline delivery of activities, 60.6% to contribute to planning and implementation of future Superbugs projects. Crucially, 30.3% of respondents even expressed an interest in using Superbugs as a platform to lead their own public engagement activities, testimony to the confidence and motivation gained from volunteering in the pop-up shop (Figure 7E). While 24.2% of respondents stated they would not like to participate in future Superbugs activities it is worth considering that this may have been affected by the fact that many pop-up shop helpers were students or staff on short-term contracts.

### 3.8 Barriers for participation in the event and potential facilitators

We recently conducted an extensive study on benefits of public engagement in research and barriers to participation, identifying that motivation for public engagement is relatively widespread amongst academic scientists and support staff but that structural and systemic challenges limit its full potential (31). Here, 23 out of 33 (69.7%) pop-up shop helpers responding to this section of the survey stated that they had experienced difficulties or limitations to their participation in the pop-up shop event. Many of these issues were venue and event-related, such as high workload on days with insufficient volunteer numbers (27.3%), problems with travel to/from the location (21.2%), cost implications (9.1%) and lack of clarity of their role (6.1%). However, helpers also highlighted a perceived lack of experience (15.2%) and resistance from their supervisor or line manager (9.1%; Table 3).

**Table 3:** Perceived barriers and facilitators for increased participation in the pop-up shop.

Questions	
Did you experience any difficulties/limitations to your participation in Superbugs?	
Lack of volunteer numbers increasing work demand	27.3%
Travel to / Location of pop-up shop	21.2%
Lack of experience in public engagement/communication	15.2%
Resistance to participation from supervisor/line manager	9.1%
Cost implications of participation	9.1%
Lack of clarity to role at Superbugs	6.1%
Other	12.1%
I did not experience difficulties or limitations to my participation	30.3%
What improvements could be made to improve your Superbugs experience / facilitate easier participation in future?	
More volunteers	48.5%
Training in science communication / public engagement	30.3%
Institutional support to participation	30.3%
Institutional recognition of participation	30.3%
Funding for travel support	27.3%
Other	3.0%
Which of the below do you feel would be an appropriate recognition of your participation in Superbugs?	
Named recognition on Superbugs website	63.6%
Certificate of participation	51.5%
Named acknowledgement on Superbugs published manuscripts	51.5%
Named authorship on Superbugs published manuscripts	30.3%
Personalised correspondence (card/email)	24.2%
Named recognition on Superbugs social media	21.2%
Volunteer appreciation event	15.2%
Superbugs-themed gift	15.2%

Difficulties/limitations to participation and suggested improvements to enhance participation in future events, as collated from a total of 33 completed post-event questionnaires. Respondents could give multiple answers.

Suggested improvements to mitigate such barriers in future deliveries of public engagement activities included funding for travel support (27.3%), training in science communication (30.3%), institutional support (30.3%), institutional recognition (30.3%), and a larger number of volunteers (48.5%). With regard to direct recognition of their participation in the pop-up shop, helpers suggested to be named on the Superbugs website (63.6%), issued a certificate of participation (51.5%), acknowledged in a publication (51.5%), or named as co-author of a publication (30.0%; Table 3). Other suggested options of recognition included personalised correspondence, named recognition in social media, Superbugs-themed gifts and a volunteer appreciation event.

Of note, some of these suggestions were directly acted upon. In particular, all helpers and contributors were named on a bespoke acknowledgement page on the Superbugs website^5^ and in the acknowledgement section of this manuscript. All helpers received an official certificate that confirmed their participation after the event. A reception with snacks and drinks was held at the end of the last day of the pop-up shop, to thank helpers for their contributions, and let them choose a small present from the shops displays as token of appreciation, such as a cuddly giant microbe or a popular science book of their choice. Helpers who made a substantial contribution to the event with regard to design, delivery and/or evaluation were named on several conference abstracts and co-authored the present manuscript, thus generating academic outputs and recognition for key participants.

### 3.9 Post-event legacy

The successful delivery of the pop-up shop led to a number of immediate follow-up opportunities for the Superbugs co-leads to present their work to specialist and lay audiences. These included invited talks and oral and poster presentations at six events organised by Cardiff University, the British Academy and the British Society for Immunology, reaching over 2,250 delegates and attendees with a predominantly professional background alongside patient representatives, service users and other lay people (Supplementary Table 7). The Superbugs team also participated in the Swansea Science Festival—the largest festival of its kind in Wales—with an interactive exhibition stand showcasing the breadth of content optimised during the earlier pop-up shop (Supplementary Table 7). An interactive workshop at a local primary school allowed us, for the first time, to exhibit parts of the horror show such as the ‘plague doctor’ and ‘Typhoid Mary’ mannequins outside the pop-up shop setting, with further schools having expressed an interest in hosting similar workshops. Finally, a webinar within Cardiff University’s Science in Health public lecture series directly based on the content of the pop-up shop horror show, with the title ‘A Microbial Horror Show: Devastating outbreaks of disease that shaped how we fight infections’, was attended by 97 people and received highly positive feedback (Supplementary Figure 18), thus reaffirming the attractiveness and effectiveness of the horror show concept and its underlying storytelling for members of the public. Taken together, these occasions allowed the Superbugs team to engage more than 7,250 professional and lay people, in addition to, and exceeding, the original audience size of 4,955 pop-up shop visitors. These numbers were amplified further through a series of reports about the pop-up shop targeting professional and public stakeholders such as Cardiff University’s Widening Participation Annual Report (32) and a summary published by the British Society for Immunology (33). A recording of the horror show lecture has been posted on YouTube and continues to attract viewers (Supplementary Table 7).

## 4 Discussion

Effective public engagement extends beyond increasing awareness and knowledge; initiatives that integrate science into everyday life through creative and participatory activities foster a culture in which science is shared, inclusive and relevant (34). The ‘science to the city’ model exemplifies this vision by translating scientific research into lived experience and public conversation. Embedding science in the spaces where people live and interact helps normalise curiosity, encourage dialogue and promote informed decision-making (35, 36). Such engagement is not just a supplementary endeavour but a critical component of addressing contemporary health challenges and improving health literacy, as exemplified by events focused on infection, AMR and vaccination (14, 22, 23, 37). The present study demonstrates that innovative models like pop-up science shops can help bridge the divide between research, healthcare and society, by transforming public spaces into arenas of discovery, reflection and empowerment (14, 29). Over a 10-week residency, we not only engaged visitors in the pop-up shop itself but also built relationships with local retailers and service providers. This ongoing presence—and becoming part of the ‘St David’s family’ for a while—likely helped reduce perceived barriers between scientists and the public further. Critically, we extend previous observations and provide evidence that such an approach is also of direct benefit to those creating and delivering educational and engaging activities in public spaces, with a positive impact on academic staff and students, personally and professionally (38).

While an increasing number of academic organisations utilises pop-up approaches to raise awareness and understanding of different areas of public interest, for example the GSI Helmholtz Centre for Heavy Ion Research in Germany,^6^ the Pop-Up Curiosity Shop of Science and Culture at the University of Exeter,^7^ the Northumbria University STEM education research group^8^ and the Swindon Imaginarium,^9^ there is only sparse literature about the short-term and long-term outcomes of such endeavours (14, 29, 39). Our evaluation shows that the Superbugs pop-up shop substantially improved understanding of key health concepts, increasing knowledge of antibiotics, AMR and vaccines while reducing common misconceptions.

It should be noted that our method of collecting data for the pre and post levels of domain understanding of visitors consisted of placing pipette tips in foil-covered laboratory bottles, which the person felt were labelled with the right answer. This was in keeping with the fun scientific flavour of the activities but it did limit our evaluation methods in that we were only able to track understanding across the group, rather than whether participants had individually improved their understanding. The benchmarking was also voluntary, with not all participants answering the questions on either entering or leaving the shop, leading to possible bias. This risk of bias was higher for the sessions involving the general public than school sessions, since in school visits there was negligible drop-out in completing the pre and post-visit benchmarking questions. Reassuringly, the learning outcomes per class of pupils matched the overall patterns across the duration of the pop-up shop event.

Visitor experience was overwhelmingly positive. Importantly, surveys showed that the impact of the event persisted for months after the visit, including sustained learning, continued conversations with friends and family, and intentions toward more responsible health behaviours. The event also significantly sparked children’s interest in science and health, supporting curiosity and complementing the school curriculum, and led to an increased awareness and uptake of comprehensive educational online resources developed earlier (30). The pop-up shop model therefore succeeded not only in communicating information but also in increasing the agency of our visitors, shifting public engagement from a deficit model to aiding citizen empowerment. As such, our approach extended previous studies and provided important insights for future deliveries aimed at converting members of the public from passive recipients of scientific messages into co-responsible actors and guardians of community health. However, it should be noted that overall response rates were modest, with only 5.2% of visitors completing the original questionnaire and only 9.0% of those returning the follow-up survey. While such low levels of voluntary engagement might be common in large-scale open public events, they limit the extent to which robust conclusions can be drawn about the learning outcomes across all visitors. Future events may benefit from stronger prompts and incentives to leave feedback and a better balance between high response, low depth surveys and optional low response, high depth follow-up.

Visitor patterns across the four-week block suggested that interactions were more dependent on passing footfall and less with social media exposure. This view is particularly supported by the unexpected correlation of visitor numbers with weather conditions, with both visitor numbers and engagement with the activities on offer tending to increase on rainy days. This finding is consistent with earlier studies indicating that rain can influence footfall, decreasing it in weather-exposed high street stores while increasing it in weather-protected shopping centres such as the location of our pop-up shop (40). However, the observed weather effects were relatively modest. Overall, the stability of visitor numbers over the four-week periods highlights the resilience and adaptability of the pop-up shop model, representing an intrinsic advantage over outdoor events that are far more dependent on external factors beyond the control of the organisers.

It was notable that social media played only a modest role in promoting this event. Although posts were shared by numerous advertisers including the Superbugs team itself as well as Cardiff University, the shopping centre, a regional broadcaster, visiting schools and various local city guides, overall interaction was low. This may have reflected factors such as the level of engagement offered by the posts themselves and/or a mismatch between the online audience and the demographic that ultimately participated. Despite the limited social media reach, in-person attendance at the event was strong, suggesting that other influences were more important in motivating people to visit Superbugs, endorsing our overarching aim to take science to the city and target unsuspecting members of the public directly on site. This dynamic will be examined more closely in future events.

Compared to a previous event that attracted 6,566 people over the first 2 weeks of the 2019 summer holidays (14), total visitor numbers in 2025 were comparatively lower, despite similar timing and location. General footfall may have been affected by the fact that in 2025 the Superbugs pop-up shop was surrounded by several units in the immediate vicinity undergoing refurbishments and/or a change in ownership, leading to this area of the mall being temporarily less busy than previously. Boosting footfall for a short time, an Oasis merchandise store that had been set up directly opposite the Superbugs pop-up shop coincided with the rock band’s highly anticipated reunion concerts in Cardiff, which constituted arguably the biggest and most popular public event in South Wales that year. While this fan shop was frequented by thousands of people, it only existed for a single week and incidentally closed the very day before the Superbugs pop-up shop opened its doors. We thus missed out on the unique opportunity to benefit from this increased footfall. But, in the words of one of the most iconic Oasis songs, we don’t look back in anger.

The concept for a horror-themed exhibition featuring emotionally and sensorially rich displays had originally been sparked by an inter-school art competition held at the beginning of 2025 (not shown), in which a striking number of submissions depicted themes related to the Black Death and the iconic attire of mediaeval plague doctors. This apparent fascination for the plague was likely a direct result of the emotional and educational impact of this gruesome part of human history on pupils, which features prominently in the Curriculum for Wales (41). Curiosity-driven demonstrations—those that are bizarre, unexpected or challenging—are consistently the ones that are the most popular, most commonly discussed and most strongly retained in both short- and long-term memory (42). The availability of a row of former fitting rooms at the pop-up shop location therefore lent itself to the creation of individual horror booths, with a life-sized mannequin dressed as plague doctor serving as central feature, alongside further exhibits evoking feelings like surprise, empathy or discomfort. By humanising disease history and personalising otherwise abstract scientific and medical concepts (43), figures such as a polio-paralysed child on crutches, an outcast child with leprosy, ‘Typhoid Mary’ and a hospital worker in full COVID-19 protective gear carried inherent emotional and moral weight. Integrating these affective responses into the learning process helped shape understanding and prompted reflection on issues such as stigma, responsibility and societal responses to illness, and provided natural opportunities for conversations with pop-up shop helpers. Encouraging visitors to compare past and present perspectives of infection control and prevention, and consider broader personal or social implications fostered rich learning experiences that combined depth of engagement with a strong element of entertainment. As such, our approach reaffirms the power of emotional and immersive learning strategies that has led to their increasing adoption in museum settings and other areas of public learning and education (44–47).

The pop-up shop had a direct impact on experiences and skills of voluntary helpers. Our own recent survey amongst academics and professional staff identified barriers and facilitators to participation in public engagement activities, highlighting the need for better institutional support, recognition and training (31). These views were matched by the present cohort of helpers who reported lacking experience and training, and—most worryingly but in line with previous studies (48–50)—resistance from their supervisors or line managers as perceived barriers for their participation. These obstacles notwithstanding, our evaluation demonstrates that events like this can directly support the personal and professional development of helpers, increasing their confidence, communication skills and general enthusiasm for public engagement, as well as a willingness to take on more responsibilities and even leadership roles in future projects. As such, we provide proof of concept that embracing a culture of public engagement offers valuable benefits both for the public and for academic staff and students (31). However, our findings are based on a single event in one location, and thus are only the beginning of a wider appreciation of public engagement as a natural part of the role of academic researchers and educators (51). Our data support earlier calls for systemic reforms to enable greater participation, including providing tailored training in science communication, sustained funding for community projects, resource and support staff, and academic frameworks that acknowledge and reward public involvement and engagement (16).

It should be acknowledged that survey-based analyses of self-reported attitudes and intentions have inherent limitations (52). A central concern is self-selection bias, as those who choose to complete questionnaires are often those who feel particularly satisfied or dissatisfied, and therefore may hold views that are not representative of the wider audience (53). In this case, the diversity of the public visiting the shop—while being one of its design strengths—resulted in a wide variation in levels of visitor engagement. Anecdotal evidence suggests that adults accompanied by children were the most engaged with the activities, which likely introduced an additional source of self-selection bias in the survey responses. Similarly, the survey of pop-up shop helpers yielded 33 responses from a total of 54 volunteers, raising the possibility of non-response bias.

Taken together, our work demonstrates that an interactive and immersive pop-up shop can serve as a powerful, inclusive and sustainable model for public engagement, with significantly improved public understanding of key health concepts, long-term changes in self-reported intentions and attitudes, and meaningful professional development for volunteers. The pop-up shop functioned as organic collision space between scientists stepping outside their academic ‘ivory tower’ and members of the public in their everyday lives. Operating as a social laboratory in a non-institutionalised and non-intimidating environment, it facilitated engagement and exchange for mutual benefit. Against the backdrop of a global crisis of growing distrust in science and in scientists—exacerbated by persisting inequalities in access to education, healthcare and representation—our approach offers a refreshing and empirically evaluated solution for rebuilding trust at the local level, a challenge shared by societies worldwide. Free entry, an attractive high-footfall location, accessibility and family-friendly design were key drivers of attendance and therefore critical to success, alongside a constructive collaboration between higher education institutions and retail and service partners. Social media appeared to play a surprisingly small role in boosting attendance, in contrast to their effectiveness in driving traffic to the Superbugs educational website (30). While it was not a formal part of the evaluation, our model also demonstrates that bilingual engagement is possible and appreciated by visitors, thereby especially enhancing the learning of children who may receive their education in a minority language, as is the case for those pupils in the UK’s devolved nations who attend Welsh, Scottish Gaelic or Irish-medium schools (30, 54, 55). This experience is likely to be transferable to other regions where a dominant scientific language may contribute to the cultural or educational alienation of diverse communities. Beyond the immediate delivery of the event, the pop-up shop led to ample opportunity for follow-on presentations to lay and expert audiences, academic outputs and public-facing reports, thus reaching further demographics and creating a lasting legacy. Our findings suggest that the pop-up shop approach can be applied and scaled to other areas of public relevance, with visitors indicating that they would readily embrace those occasions and visit scientific pop-up shops with themes as varied as health, diet, pollution and space. Similarly, we see no reason why the concept could not be adapted to other cultural and socioeconomic contexts, including settings with restricted resources. We conclude that non-academic spaces with high natural footfall present an ideal setting to engage diverse demographics in an entertaining and playful but memorable and impactful way, transcending the boundaries of conventional academic engagement.

## Data Availability

The original contributions presented in the study are included in the article/Supplementary material.

## Footnotes

1
www.superbugs.online/pop-up-2025

2
www.superbugs.online

3
http://meteostat.net

4
www.superbugs.online/pop-up-2025/body-swabs

5
www.superbugs.online/pop-up-2025/acknowledgements

6
www.gsi.de

7
www.exeter.ac.uk/research/publicengagement

8
http://nustem.uk/popupshop

9
www.festivaloftomorrow.com/our-projects-and-news/imaginarium
