# Supplementary material for: Impact of a large-scale interactive and immersive science pop-up shop about infection and hygiene on visitors and volunteers
Source: Front Public Health. 2026 Apr 23;14:1812530. doi: 10.3389/fpubh.2026.1812530 (PMC13149357; doi:10.3389/fpubh.2026.1812530)
Supplement: Supplementary file 1 [file Data_Sheet_1.PDF]

Impact of a large-scale interactive and immersive science pop-up shop about infection and hygiene on visitors and volunteers

Jonathan M. Tyrrell, Abdulsalam Abdulgafar, Pouya Sadeghzadeh Rahimi, Georgina B. Sparling, Friday K. A. Cox, Lila-Blythe Maros, Jeff Lewis, Naomi Stanton, Sarah Hatch, Carl Smith, Simone M. Cuff, Rebecca J. Aicheler, Matthias Eberl

SUPPLEMENTARY INFORMATION

Supplementary Materials and Methods

- Shop location
- Content, materials and exhibits
- Infoboards
- Main activities
- Sticker challenge
- Treasure hunt
- Horror show
- Other major features of the pop-up shop

Supplementary Tables

- Suppl. Table 1. Recommended popular science books on display in the pop-up shop's reading corner.
- Suppl. Table 2. Selection of social media posts referring to the 2025 pop-up shop.
- Suppl. Table 3. Qualitative feedback from pop-up shop visitors as extracted from the post-event questionnaires.
- Suppl. Table 4. Demographics of pop-up shop visitors completing the long-term impact survey.
- Suppl. Table 5. Qualitative feedback from pop-up shop visitors as extracted from the long-term impact survey.
- Suppl. Table 6. Demographics of pop-up shop helpers completing the post-event survey.
- Suppl. Table 7. Presentations by the Superbugs co-leads showcasing the learnings from the pop-up shop to diverse audiences.

Supplementary Figures

- Suppl. Fig. 1. Pop-up shop front.
- Suppl. Fig. 2. Word clouds summarising feedback by visitors of the 2019 event.
- Suppl. Fig. 3. Superbugs infoboards.
- Suppl. Fig. 4. Main shop activities for the sticker challenge.
- Suppl. Fig. 5. Sticker challenge.
- Suppl. Fig. 6. Treasure Hunt activity.
- Suppl. Fig. 7. Superbugs Horror Show displays.
- Suppl. Fig. 8. Additional shop features.
- Suppl. Fig. 9. School art exhibition.
- Suppl. Fig. 10. Arts and crafts corner.
- Suppl. Fig. 11. Visitor engagement rates.
- Suppl. Fig. 13. Correlation between daily pop-up shop activities and the weather.
- Suppl. Fig. 13. Quantitative and qualitative impact evaluation.
- Suppl. Fig. 14. Assessment of learning outcomes for a total of six school visits.
- Suppl. Fig. 15. Assessment of learning outcomes for a total of six school visits.
- Suppl. Fig. 16. Long-term impact of the event.
- Suppl. Fig. 17. Quantitative feedback from helpers in the pop-up shop.
- Suppl. Fig. 18. Feedback by attendees of the public lecture 'A Microbial Horror Show – Devastating outbreaks of disease that shaped how we fight infections'.

SUPPLEMENTARY MATERIALS AND METHODS

SHOP LOCATION

The pop-up shop was set up in a vacant retail unit on the upper level of the St David's Dewi Sant shopping centre in Cardiff (UK). With a yearly footfall of approx. 30 million people, St David's is one of the UK's busiest shopping centres, right in the heart of the Welsh capital. The unit was situated in the most spacious and busiest part of the shopping centre, directly next to the John Lewis store and near other popular shops (*e.g.* Zara, Apple), family-friendly restaurants (*e.g.* Nando's, Pizza Express), the Treetop Adventure Golf experience, and the elevators and stairwells to the main car park.

The unit had previously been used by Co.Lab, a dynamic pop-up retail concept that had hosted independent fashion, beauty and lifestyle brands, alongside community events like workshops, styling, yoga and wellness sessions—an ideal space to accommodate an immersive public engagement event spanning different activities, with each part of the unit painted in different warm colour tones. A wooden floor, wooden beams inside the shop and an inviting entrance with a wooden lattice between four large shop windows added to the welcoming nature of the unit (Suppl. Fig. 1). The total usable area was approx. 891 m^2, including eleven former fitting rooms in the back of the shop; in addition, the Superbugs team had access to extensive storage space upstairs, a former staff room, a staff kitchen and three staff toilets. In total, Superbugs occupied this retail unit for ten weeks, including four weeks of setting up content and activities, four weeks of in person delivery, and two weeks of dismantling the shop.

CONTENT, MATERIALS AND EXHIBITS

Bilingual infoboards, a large backlit banner behind the swabbing station, a large Superbugs logo above the shop entrance and sticky germ-shaped floor footprints were produced by Morgans Consult. Smaller posters were printed by the staff printing services at Cardiff University and Swansea University. Materials and props for the shop were purchased from local or online retailers including Amazon, B&M, Boots, Dunelm, eBay, IKEA, John Lewis, Jokeshop, Morrisons, One Beyond, Screwfix, Superdrug, Tesco, The Range, The Works and local charity shops. Other materials such as laboratory items, personal protective equipment, surgical scrubs and display items were provided free of charge by academic and clinical colleagues and facilitators (see Acknowledgements for full list). Furniture such as tables, chairs, plinths, sofas, shelves and a stage as well as mannequins and a large fridge were kindly provided free of charge for the duration of the event by St David's and John Lewis.

Glitter Bug Potion was purchased from Food Safety Direct, Wash & Glow UV Germ Lotion and UV boxes from Glowtec. Columbia blood agar plates were purchased from Public Health Wales. Superbugs-branded merchandise was purchased from Total Merchandise and Get Yourself Noticed, cuddly bacteria and viruses from GIANTmicrobes. Microscopes were kindly provided by the teaching labs of Cardiff University's Medical Pharmacology programme. Popular science books were kindly donated by some of the authors, others were purchased used from Amazon. Free literature for visitors to take home was kindly provided by the British Society for Immunology (vaccination guides) and Cardiff University's School of Medicine (Science in Health programme); Cardiff University, Cardiff Metropolitan University and Swansea University provided information about their medical or biomedical degrees targeted at GCSE and A Level pupils.

INFOBOARDS

Wall-mounted large-scale Foamex infoboards of 90×87 cm in size provided the scientific background for the pop-up shop activities, spanning six major themes (Suppl. Fig. 3). 'A truly microbial world' gave an introduction into microbial organisms and where to find them, accompanying the swabbing station and a display of environmental and commensal microbes grown on agar plates. 'A microscope world' explained the different types and shapes of microbes, and what they look like under a microspore, providing context for the microscope station and a display of cuddly microbes. 'Infections' at the entrance of the horror show provided context on how pathogens cause disease and how infections spread. 'Antimicrobial resistance' complemented a tin can alley activity to learn about antibiotics and how bacterial can become resistant to them. 'The spread of resistance' explained the science behind a plasmid ring toss game and showed how bacteria can pass on AMR genes to each other. 'Vaccines' next to a large ball pit gave an introduction into the cells of the immune system and how vaccines work. All infoboards were designed in an accessible way, combining cartoons, photos and infographics, and were displayed side by side in an English and a Welsh version.

MAIN ACTIVITIES

1. **Grow your own microbes.** An opportunity for visitors to appreciate that microbes are literally everywhere, by swabbing body parts or personal items (Suppl. Fig. 4A). Samples were taken using cotton buds, or simply by leaving fingerprints, in order to grow microbes on blood agar plates. Visitors turned out to be highly creative and took swabs from their tongues, ears and noses, feet and hands, belly buttons and armpits, mobile phone screens, jewellery and much more—some of them were even brave enough to kiss the plates! No identifiable information was taken. Agar plates were then incubated by the Superbugs team at room temperature for 2–3 days, with all necessary safety precautions in place. Anonymised photos of each plate were then posted on the Superbugs website at *https://www.superbugs.online/pop-up-2025/body-swabs*, where visitors could view the results. A display next to the swabbing station showed many examples of body and environmental samples previously grown by the Superbugs team (Suppl. Fig. 4C).

2. **Hand-washing.** An activity to teach good hygiene, by using fluorescent gel that 'magically' makes hands glow in the dark (Suppl. Fig. 4C, Suppl. Fig. 4D). GlitterBug Potion and UV Germ Lotion are commercial products that make hand hygiene education interesting, fun and memorable. They are fluorescent hand lotions that, when used with a UV lamp, make it possible to see how well our hands are washed, and to visualise contamination and illustrate how germs can spread. This activity is equally suited for schools and public events, as well as for the training of healthcare professionals and people handling food.

3. **Microscopes.** An activity to get close up with different micro-organisms and appreciate their size and their variety (Suppl. Fig. 4E, Suppl. Fig. 4F). The station featured four microscopes where visitors could explore fixed slides with the following microbes: *E. coli* as an example of a Gram-negative bacterium; *Staphylococcus aureus* as an example of a Gram-positive bacterium; the mould (and antibiotic producer) *Penicillium*; and the freshwater protist *Paramecium*. This activity was combined with a colourful and playful display of cuddly 'giant microbes' illustrating the enormous variety of micro-organisms (Suppl. Fig. 8D). The display covered bacteria such as *E. coli*, *Staphylococcus aureus*, *Streptococcus pyogenes* and *Clostridioides difficile*; viruses including rhinovirus, Ebola and swine flu; protozoa such

as *Plasmodium falciparum* and *Giardia lamblia*; and the black mould, *Stachybotrys chartarum*.

4. **Vaccine ball pit.** This activity was aimed to explain how the immune system detects and fights 'bad germs' (a spiky ball) amongst all other structures that our body is constantly in contact with (Suppl. Fig. 4G, Suppl. Fig. 4H). This could either be to explain what happens when a pathogen invades the body during an infection and is recognised and attacked by immune cells, or to demonstrate the effect of vaccination when the body produces specific antibodies and immune cells protecting us from 'bad germs'. In each case, players simply had to find a spiky ball amongst 1,500 normal balls. Sounds easy—but not when you are blindfolded.

5. **Space lab.** This fully immersive virtual reality experience brought biomedical science to life, showcasing the vital role biomedical scientists play in modern healthcare (Suppl. Fig. 4I). The Institute of Biomedical Science (IBMS)'s Accredited Space Lab is designed to spark curiosity, break down barriers and inspire the next generation of healthcare professionals. Set inside a futuristic 'space lab', users were guided through an interactive journey, taking control of a simulated urine sample and following it through each stage of microbiological testing. From sample collection to result analysis, participants got a close-up look at the processes behind diagnosing illness. This was not just a virtual experience—it was an invitation to explore what biomedical scientists do and why their work matters, and offered visitors a chance to see if a career in biomedical science might be right for them.

6. **Antimicrobial resistance tin can alley.** This activity was to raise awareness of the consequence of AMR for treating bacterial infections. Using 'antibiotic' bean bags, players first had to knock over a pile of cans representing susceptible bacteria, to learn about the effectiveness of antibiotics in treating infections. Next, a facilitator or co-player waved a large shield in front of the cans, demonstrating the difficulty of treating an infection caused by resistant bacteria (Suppl. Fig. 4J). Curiously, some players quickly discovered that working as a team and throwing multiple bean bags at the same time overcame the person waving the shield—thus mimicking current approaches of treating critically ill patients with a combination of several antibiotics at the same time.

7. **Plasmid ring toss.** Bacteria have a unique way of passing on traits to neighbouring cells of the same, or even other species, by being able to transfer small rings of DNA (plasmids) containing genetic information such as antibiotic resistance genes. To understand how AMR spreads, players had to throw plastic rings at a large-scale bacterial model (Suppl. Fig. 4K, Suppl. Fig. 4L). Even if players had never heard of plasmids before, or even of DNA and genes, they easily grasped the concept of how bacteria can send rings of 'information' to each other.

STICKER CHALLENGE

To encourage maximum engagement with the pop-up shop content, visitors could collect colourful stickers representing fictious 'germs' at each of the seven stations outlined above. Visitors completing at least six activities and presenting their activity cards with six different stickers at the welcome desk could choose from a range of Superbugs-branded small prizes that included pens, keyring torches, rulers, bookmarks, wristbands, water bottles and balloons as reward. This concept had already been successfully used at a previous event (Tyrrell et al.,

2022) and not only motivated visitors to explore all activities but also allowed a simple tracking of the level of engagement, by counting the number of completed sticker challenges. Given the wide age range of visitors, we decided to offer a total of seven activities to allow for more flexibility according to the age of the visitors—taking into account that the VR headset activity was mainly targeted at secondary school children and adults, while the ball pit was more suitable for toddlers and primary school children (many visitors actually did all seven activities regardless). Most importantly, the back of the sticker card depicted a playful certificate as 'Antibiotic Resistance Champion' (Suppl. Fig. 5), providing a simple but memorable summary of five steps that every person can do to contribute to good antibiotic steward ship and help fight the spread of AMR.

TREASURE HUNT

This activity combined a popular activity mainly targeted at school age children with educational content, as developed by us earlier (Tyrrell et al., 2024). The task was to find a total of six 'nasty germs' that had 'escaped' and were 'hiding' somewhere in the shop, ranging from a couple of relatively easy locations near the welcome desk to more challenging locations—including within the horror show (Suppl. Fig. 6). Younger visitors were simply tasked to find the hidden germs, older ones were encouraged to write down their scientific names and some basic information about the infectious diseases they cause. Unexpectedly, this activity was readily taken up even by adult visitors, as a fun way to learn interesting facts about infections while being encouraged to explore the entirety of the large pop-up show area. Similarly to the sticker challenge, players who completed their treasure hunt sheet could choose from a range of Superbugs-branded small prizes at the welcome desk (Suppl. Fig. 4B).

HORROR SHOW

General concept. The retail unit available for the Superbugs pop-up shop included a spacious and secluded area with a total of 11 former fitting rooms. Using posters, mannequins and props, each cubicle was converted into a bespoke display around an infectious disease of global importance. Combining scary and informative content in an immersive 'horror show', we aimed to inspire visitors by showing the stunning advances in science and health that have improved the diagnosis, treatment and prevention of life-threatening infections. All written content was provided bilingually in English and Welsh. After the event, the materials used for the pop-up horror show were made available on the Superbugs website at *https://www.superbugs.online/pop-up-2025/horror-show*; some aspects of the horror show (including a mannequin dressed as medieval plague doctor) have already been re-used for various in-person events such as a visit to a local primary school and a public involvement and engagement showcase event at Cardiff University, and as foundation for a public lecture (Suppl. Table 4).

Shop entrance. The front entrance of the pop-up shop featured a mannequin dressed as hospital doctor in scrubs, facemask, goggles, gloves and plastic apron, holding a giant cuddly bacterium and inviting visitors to come in and explore the shop further (Suppl. Fig. 7A). Engaging signs throughout the pop-up shop led visitors to the hidden horror show situated in the back of the shop. The start of the actual horror show was marked by a real-life skeleton sitting in a director's chair (Suppl. Fig. 7B).

1. **Antimicrobial resistance – The end of modern medicine?** Bacteria, viruses, fungi and parasites are evolving to survive drugs that were designed to kill them. Overuse and misuse of antibiotics in healthcare and agriculture are accelerating this process. The display featured a stand showing the outer packaging of antibiotics and antivirals, a wall covered with patient information leaflets for different antibiotics, and a plastic apron worn by hospital workers as protection against the spread of resistant bacteria (Suppl. Fig. 7C). Posters provided further context about AMR, and a timeline of antibiotic discoveries and the emergence of resistant strains. The entrance to the booth featured a gigantic cuddly representation of methicillin-resistant *Staphylococcus aureus* (MRSA).

2. **Measles and polio – Preventable childhood diseases.** Before vaccines became widely available, childhood infections like measles and polio were common. These illnesses can cause serious health problems and may lead to long-term disability or even death. The display featured a child mannequin wearing a pyjama standing next to his bed on crutches and looking at himself in a mirror, recreating a historical photo of a boy suffering from polio-induced paralysis (Suppl. Fig. 7D). The walls of the cubicle were covered in original packaging of commonly used vaccines, and there were free copies of the British Society for Immunology's 'Childhood Vaccination Guide'. Posters gave background about measles and polio as examples of vaccine-preventable infections, including graphs illustrating the dramatic drop in measles and polio cases soon after the introduction of safe and effective vaccines. A further poster contained a public letter written by the author Roald Dahl after his daughter Olivia had died from measles in 1962, highlighting the seriousness of the disease and advocating for the use of the measles vaccine.

3. **Guinea worm – Parasitic worms.** Guinea worm is a parasite endemic in parts of Asia and Africa, which lives in lakes and stagnant waters, carried by microscopic water fleas as intermediate hosts. It infects mainly humans but also some wild and domestic animals. The display featured a water pot covered with mesh to illustrate the effectiveness of filtering drinking water before consumption (Suppl. Fig. 7E). Posters and photos of patients and communities in affected areas illustrated how worms are extracted from patients (and a dog), and how simple measurements have succeeded in reducing the prevalence of the disease dramatically. A drawing of a human body on the booth's mirror showed the life cycle of the worm. A free copy of a WHO cartoon explaining the disease in simple but effective terms was available for visitors to look at and to take home.

4. **Smallpox – The disease that made history.** Smallpox was a highly contagious and deadly disease caused by the variola virus. It spread through contact with infected individuals, typically via droplets when patients coughed, sneezed or simply talked. The display featured an inflatable globe labelled with the words "smallpox is dead" across, recreating the iconic front title of the WHO magazine's May 1980 issue (Suppl. Fig. 7F). Posters provided information about the disease in general, a summary of the history of the smallpox vaccine, a map illustrating when smallpox was eradicated in different parts of the world, and a photo of Ali Maow Maalin—the last person ever to contract natural smallpox. The display also featured several free copies of a story about Edward Jenner's smallpox vaccine, written by a Welsh year 9 pupil as part of an inter-school competition, for visitors to take home.

5. **Typhoid – Asymptomatic carriers.** Typhoid fever is a serious infectious disease caused by the bacterium *Salmonella enterica* serotype Typhi. It spreads primarily through contaminated food and water, making it common in areas with poor sanitation. The display featured a mannequin dressed up as the Irish cook Mary Mallon preparing miniskulls in a frying pan, recreating the infamous illustration in the 1909 New York American article

labelling her as 'Typhoid Mary' and accusing her of spreading typhoid (Suppl. Fig. 7G). Posters provided information about the disease in general, and about the role of asymptomatic carriers not only with regard to typhoid but also other infectious diseases.

6. **Plague – The Black Death.** Transmitted by rat fleas, this disease caused by the bacterium *Yersinia pestis* led to several devastating pandemics throughout history. The display featured a mannequin dressed up as a medieval plague doctor standing next to a dead child, with a black rat illustrating the importance of pests as carriers of the disease (Suppl. Fig. 7H). Posters provided information about the disease in general and an explanation of the different parts of a plague doctor's attire as an early form of personal protective equipment.

7. **Malaria – Insect-borne infections.** Malaria is a life-threatening disease caused by tiny parasites, transmitted to humans through the bite of female *Anopheles* mosquitoes. The display featured a cage with approx. 100 harmless *Culex pipiens* mosquitoes as centre piece (kept alive throughout the event by feeding on sugar water), alongside examples on how to protect oneself against insect bites, using insect repellent (Suppl. Fig. 7I). The entrance to the cubicle was covered in a large mosquito net that visitors had to walk through to access the display. Posters provided background about malaria, the life cycle of the parasite and a close-up view of a mosquito's head. An additional poster illustrated the role of vectors in transmitting diseases, covering mosquitoes, tsetse flies, fleas, ticks and other species.

8. **Cholera – The beginning of epidemiology.** Cholera is a deadly infectious disease caused by the bacterium *Vibrio cholerae*, which spreads through contaminated water or food. It causes acute diarrhoea, leading to rapid dehydration and, if untreated, death. The display featured a jar of clean drinking water and one with dirty, 'contaminated' water to illustrate the importance of sanitation and hygiene in containing cholera and other diseases—next to a reminder that according to the World Health Organisation 2.2 billion people worldwide currently lack access to clean drinking water (Suppl. Fig. 7J). Giant microbes were used to showcase a variety of diarrhoea-causing bacteria (*Vibrio cholerae*, *Shigella dysenteriae*, *E. coli*, *Campylobacter jejuni*, *Listeria monocytogenes*). Posters provided information about cholera, an account of John Snow's epidemiological work and his 'ghost map' of London Soho from 1854, historical public health warnings about cholera outbreaks, and a map of current cholera cases in the world.

9. **Leprosy – Medieval beliefs versus modern medicine.** Leprosy is an ancient disease that has haunted humanity for much of its history. It is caused by *Mycobacterium leprae* and spread by coughs and sneezes. The display featured a mannequin dressed up in a medieval gown, standing next to jars illustrating medieval beliefs of where the disease originated from and how to diagnose and treat it (Suppl. Fig. 7K). Posters introduced the disease itself and contrasted ancient beliefs and myths against the advances of modern science and medicine.

10. **Wound infections – Tetanus, sepsis and medicinal maggots.** Wound infections occur when harmful microorganisms invade a break in the skin or underlying tissues, leading to delayed healing and potential complications. The display featured a mannequin dressed as a hospital nurse attending to patients with different kinds of (artificial) healing and infected wounds (Suppl. Fig. 7L). Posters explained the importance of treating wound infections, a range of causative pathogens and how to target them with antibiotics, and the concept of using medicinal maggots in cleaning difficult to treat wounds.

11. **Ebola and COVID-19 – Bats as carriers of deadly viruses.** Fruit bats are fascinating and harmless animals, but they can carry viruses that can cause severe disease, without

showing signs themselves. The display featured a mannequin dressed in full protective gear as would be used by a UK hospital worker caring for a patient with a highly transmissible infection—PPE items included face shield, fitted mask with filter, hood, surgical gown, surgical gloves and disposable overshoes (Suppl. Fig. 7M). The cubicle was decorated with cuddly models of a SARS-CoV-2 virus and fruit bats as disease carriers, and the contents of a COVID-19 self-testing kit; a poster gave some background about serious illnesses being transmitted by bats, such as Ebola and—possibly—COVID-19.

Bonus display: Infection diagnosis – Detecting and treating common diseases. Infections manifest themselves in many different ways and give rise to a large number of unspecific and often overlapping symptoms, making accurate and timely diagnosis a challenge for GPs and hospital doctors. The display in the anteroom to the actual horror show cubicles featured a mannequin dressed as a hospital nurse in green hospital scrubs, and another mannequin wearing protective gear typical for a category 3 safety level laboratory (Suppl. Fig. 7N). Posters explained symptoms of common infections such as pneumonia and urinary tract infections, alongside information about nature of the causative pathogens, how to detect them using blood and urine test as well as chest X-rays, and how to treat them using antibiotics.

Bonus display: Friendly germs – Microbes producing our food. Many people think of microbes only as harmful germs that cause disease. But in reality, not all microbes make us sick. In fact, many are beneficial and play important roles in our daily lives. The display placed just outside the entrance of the horror show featured shelves with a range of food items that are produced with the help of bacteria, yeasts and moulds—ranging from bread and dairy products such as yoghurt, kefir and cheese to fermented vegetables, soy sauce, beer, wine and vinegar (Suppl. Fig. 7O). Posters gave more examples of ‘friendly germs’ producing our food and illustrated their role in providing the ingredients needed for pepperoni pizza!

OTHER MAJOR FEATURES OF THE POP-UP SHOP

The pop-up shop boasted a number of additional features, in addition to the major activities outlined above. These included colourful and inviting digital and print adverts across the shopping centre to raise awareness for the event (Suppl. Fig. 8A, Suppl. Fig. 8E). A large backlit Superbugs banner that was visible from everywhere in the pop-up shop provided an engaging background for the swabbing station—and as backdrop for selfies (Suppl. Fig. 8B).

Wall of Fame – School art exhibition. A large empty wall in the pop-up shop was converted into an art gallery showcasing drawings and written work produced by pupils at secondary schools across Wales, including the Welsh-medium schools Ysgol Llanhari (Llanharry), Ysgol Gyfun Rhydywaun (Penywaun), Ysgol Syr Thomas Jones (Amlwch) and Ysgol Syr Hugh Owen (Caernarfon), and the English-medium school St John’s College (Cardiff) (Suppl. Fig. 8C, Suppl. Fig. 9. A selection of the school art displayed during the event was later made available online in the ‘Superbugs Hall of Fame’ at *www.superbugs.online/uploads*.

Displays. A series of displays across the pop-up shop provided further context and depth. These included items such as large cuddly microbes visualising the variety of microbial organisms (Suppl. Fig. 8D), a selection of small laboratory equipment (Suppl. Fig. 8H), and mannequins dressed as laboratory scientists or healthcare professionals. A large bacterial model at the exit of the shop served as ‘thought tree’ for visitors to leave post-it notes with comments and feedback (Suppl. Fig. 8I).

Live stage. The far end of the shop featured a live stage for Superbugs researchers to give presentations and as an opportunity for visitors to ask questions (Suppl. Fig. 8F, Suppl. Fig. 8G). This was greatly appreciated during school visits where the stage was used both to give an introduction into the main themes of the event and to host fun quizzes at the end of the visit (Suppl. Fig. 15), as well as Q&A sessions about shop content or science in general—even career advice for pupils interested in science, healthcare and medicine.

Arts & crafts corner. A dedicated and brightly lit area at the front of the shop served as arts & crafts corner, providing arts supplies such as pens, pencils, colouring pens, crayons, glue sticks, pompoms and googly eyes, a range of colouring sheets and plain paper (Suppl. Fig. 8J). Representative examples produced by shop visitors were displayed around the arts & crafts corner or directly in the shop windows, thereby inviting passers-by to enter the shop (Suppl. Fig. 10). This activity was particularly attractive for young children who were too small to engage with the more advanced content of the shop but was also readily taken up by older children and teenagers. A selection of the artwork created during the event was later made available online in the 'Superbugs Hall of Fame' at *www.superbugs.online/uploads*.

Reading corner. A comfortable sitting area in an open space in the centre of the shop invited visitors to take a break and relax (Suppl. Fig. 8K). A bookshelf with recommended popular science books, some of them donated by the actual authors (Suppl. Fig. 8L, Suppl. Table 1), aimed to spark further curiosity beyond the pop-up shop content. In addition, free literature was made available that was kindly provided by the British Society for Immunology, such as the comprehensive 'Guide to childhood vaccinations' and the 'Guide to vaccinations for adults over 65' compiled by experts in the field. Cardiff University's School of Medicine provided flyers advertising its public-facing Science in Health programme. Lastly, the admission teams at Cardiff University, Cardiff Metropolitan University and Swansea provided booklets about their degree programmes for prospective students interested in applying for a study place in a biomedical or healthcare subject at one of the three universities.

Further activities. LEGO models of enzyme/substrate interactions provided a playful way to learn the action of antibiotics on target structures inside bacteria. A playdoh version of the Bristol Stool Chart explained what doctors can learn about our health from the composition of our faeces, to help diagnose digestive and infectious conditions. A fun activity with water spray bottles demonstrated how some bacterial species can form biofilms that make antibiotic treatment challenging. QR codes encouraged visitors to complete a questionnaire about their views on the pop-up shop experience and to explore specific sections of the Superbugs website.

REFERENCES

Tyrrell JM, Conlon C, Aboklaish AF, Hatch S, Smith C, Mathias J, Thomson K, Eberl M. Superbugs: raising public awareness of antimicrobial resistance through a pop-up science shop. *Research for All* 2022; **6**: 1-21.

Tyrrell JM, Ayanikkad HU, Nalleppillil-Gopakumar V, Oyebode R, Nnamdi Blessing C, Hatch S, Eberl M. Combining postgraduate research training, public engagement, and primary school science education — a Superbugs Master (MSc) class. *Frontiers in Microbiology* 2024; **15**: 1380045.

SUPPLEMENTARY TABLES

Supplementary Table 1. Recommended popular science books on display in the pop-up shop's reading corner.

Author	Title
Older audiences	
Martin Blaser	*Missing microbes*
Daniel M. Davis	*The secret body*
Andrew Doig	*This mortal coil*
Rob Dunn	*The wild life of our bodies*
Sarah Gilbert, Catherine Green	*Vaxxers*
Ben Goldacre	*Bad Science*
Adam Kay	*This is going to hurt*
Claas Kirchhelle	*Typhoid*
Raina MacIntyre	*Vaccine nation*
Stanley Maloy, Roberto Kolter	*Microbes and evolution*
Matt Morgan	*One Medicine*
Matt Morgan	*Critical*
Luke O'Neill	*Keep calm and trust the Science*
Luke O'Neill	*Humanology*
Luke O'Neill	*Never mind the b#ll*cks, here's the science*
Tom Phillips, Jonn Elledge	*Conspiracy*
Markus Schmidt	*The Art of Antibiotics*
Irwin W. Sherman	*Twelve diseases that changed our world*
Devi Sridhar	*Preventable*
John Tregoning	*Infectious*
Lucy Ward	*The Empress and the English Doctor*
Younger audiences	
Idan Ben-Barak, Julian Frost	*Do not lick this book*
European Federation of Immunological Societies	*Your amazing immune system*
Emily Grossman, Maggie Li	*Meet the microbes*
Tom Ireland	*The good virus*
Sheddad Kaid-Salah Ferrón, Eduard Altarriba	*My first book of microbes*
Ben Martynoga, Moose Allain	*The virus*
Lindsey Millar, Vivien Sarkany	*Wonderful world of the small*

Supplementary Table 2. Selection of social media posts promoting the 2025 pop-up shop. Numbers of likes, shares, views and comments as of 13 January 2026.

	**Link**	**Date**	**Reach**
**Facebook**			
St David's Dewi Sant	https://www.facebook.com/stdavidscardiff/posts/pfbid0313Y4ZU6r654uKM2ibCGjhUuymvEUgBsf44znagQZBuHgZaTiaDGqpkgPzuxNxeDDl	01/06/2025	32 likes, 13 shares, 14 comments
Superbugs	https://www.facebook.com/permalink.php?story_fbid=pfbid031PQtEMTq3q1geeZNeqDp37xRPfgfgFh2K5rsRL8JNfbmzdAdY7G26mJsyXhUcNQVl&id=61578875034466	26/07/2025	2 likes, 5 shares
Cardiff University	https://www.facebook.com/share/r/16jPvniv5L/	29/07/2025	23 likes, 2 shares
Superbugs	https://www.facebook.com/permalink.php?story_fbid=pfbid03LzZHaBmRASm6wqPYuC7UgHo72snDFXHvWRTiLgxBBo5H52XveTJFqg3pFKNTVgvl&id=61578875034466	30/07/2025	1 like, 13 shares
St David's Dewi Sant	https://www.facebook.com/stdavidscardiff/posts/pfbid02o4rH74bLtTnVqz7RkHqczAyfTQ2NsyFz5HMpX7w3ouZuSWJpcnJXZLrFTcfyg19jl	01/08/2025	6 likes, 6 shares
**Instagram**			
St David's Dewi Sant	https://www.instagram.com/p/DKhK2RFuyK8	05/06/2025	93 likes, 3 comments
St Martin's School	https://www.instagram.com/p/DL5S5m2ocot	09/07/2025	44 likes
ITV Wales	https://www.instagram.com/reel/DL-KJj5NJVL/	11/07/2025	236 likes, 3 comments
Gwyddoniaeth Llanhari	https://www.instagram.com/reel/DL-Ag-cifDN	11/07/2025	30 likes
Gwyddoniaeth Llanhari	https://www.instagram.com/p/DL-UtnPC__b	11/07/2025	27 likes
Gwyddoniaeth Llanhari	https://www.instagram.com/p/DL-XCFSCOyl	11/07/2025	48 likes
Whitchurch High School	https://www.instagram.com/p/DMLO3uLCKlM	16/07/2025	16 likes, 1 repost
Visit Cardiff	https://www.instagram.com/p/DMhs1aLMHlw/?hl=en	25/07/2025	14 likes
Cardiff University	https://www.instagram.com/reel/DMsPW2utnPk	29/07/2025	358 likes, 8 comments, 38 shares
Cardiff University (Welsh)	https://www.instagram.com/reel/DMscNOMtUQV	29/07/2025	21 likes, 2 shares
St David's Dewi Sant	https://www.instagram.com/reel/DMz9e7ONUvS	01/08/2025	54 likes, 2 shares

**X/Twitter**			
Cardiff Passport to the City	https://x.com/Passport2City/status/1943317293829681181	10/07/2025	1 like, 1 reposts, 255 views
Cardiff Passport to the City	https://x.com/Passport2City/status/1943618786726228142	11/07/2025	2 likes, 2 reposts, 121 views
**TikTok**			
Superbugs	https://www.tiktok.com/@prof_superbugs/video/7505136425470266646	16/05/2025	9 likes, 933 views
Superbugs	https://www.tiktok.com/@prof_superbugs/photo/7511401091880275222	02/06/2025	2 likes, 1933 views
Superbugs	https://www.tiktok.com/@prof_superbugs/video/7512388208789949718	05/06/2025	6 likes, 612 views
Superbugs	https://www.tiktok.com/@prof_superbugs/video/7516609693033270550	16/06/2025	9 likes, 2 pins, 743 views
Superbugs	https://www.tiktok.com/@prof_superbugs/video/7521072030817570070	28/06/2025	3 likes, 257 views
Superbugs	https://www.tiktok.com/@prof_superbugs/video/7524411925040516374	07/07/2025	1 like, 771 views
Superbugs	https://www.tiktok.com/@prof_superbugs/video/7526520236405755158	13/07/2025	6 likes, 1 pin, 775 views
Superbugs	https://www.tiktok.com/@prof_superbugs/video/7529596613061397782	21/07/2025	27 likes, 3 pins, 839 views
Cardiff University	https://www.tiktok.com/@cardiffuni/video/7532508945357933847	29/07/2025	39 likes, 8 shares, 2 pins
**LinkedIn**			
Rachael Barton	https://www.linkedin.com/posts/rachaelmbarton_superbugs-superbugs2025-outreach-activity-7358099614262009857-zqDr	07/2025	58 likes, 6 comments, 3 reposts
Cardiff University	https://www.linkedin.com/posts/cardiff-university_cardiffuniversity-prifysgolcaerdydd-activity-7355514654850203649-Rs-T	22/07/2025	65 likes, 4 comments, 9 reposts
Carl Smith	https://www.linkedin.com/posts/carl-smith-4ab604102_volunteering-superbugs-science-activity-7356716279874920449-sUmm	07/2025	46 likes, 1 comment, 3 reposts
Melika Nomiri	https://www.linkedin.com/posts/melika-nomiri-508a29298_superbugs-activity-7371608607345516544-dDQt	08/2025	9 likes, 1 comment
Natalie Joseph-Williams	https://www.linkedin.com/posts/natalie-joseph-wiliams-755766120_helping-out-at-the-superbugs-pop-up-today-activity-7348755594247319554-ZgI8	09/07/2025	36 likes, 4 reposts
**Bluesky**			
Superbugs	https://bsky.app/profile/superbugs.bsky.social/post/3lnmpoq4hgk2h	25/04/2025	4 likes, 4 reposts
Superbugs	https://bsky.app/profile/superbugs.bsky.social/post/3lnxvxmq7ok2k	29/04/2025	13 likes, 9 reposts
Superbugs	https://bsky.app/profile/superbugs.bsky.social/post/3lnzwmoadvs2g	30/04/2025	4 likes, 5 reposts
Superbugs	https://bsky.app/profile/superbugs.bsky.social/post/3loldb6i4wk2w	07/05/2025	13 likes, 7 reposts

Superbugs	https://bsky.app/profile/superbugs.bsky.social/post/3lpcmrlfufc27	16/05/2025	17 likes, 6 reposts
Superbugs	https://bsky.app/profile/superbugs.bsky.social/post/3lpcmrnbtdk27	16/05/2025	9 likes, 4 reposts
Superbugs	https://bsky.app/profile/superbugs.bsky.social/post/3lpcyjtsqhk2y	16/05/2025	6 likes, 4 reposts
Superbugs	https://bsky.app/profile/superbugs.bsky.social/post/3lqnb7t2bp22d	02/06/2025	23 like, 12 reposts
Superbugs	https://bsky.app/profile/superbugs.bsky.social/post/3lqtxtjaugc2z	05/06/2025	9 likes, 5 reposts
Superbugs	https://bsky.app/profile/superbugs.bsky.social/post/3lrsudq3sb22i	17/06/2025	16 likes, 3 reposts
Superbugs	https://bsky.app/profile/superbugs.bsky.social/post/3lrxluqqils23	19/06/2025	7 likes, 5 reposts
Superbugs	https://bsky.app/profile/superbugs.bsky.social/post/3ls6s5omyzc2s	22/06/2025	2 likes
Superbugs	https://bsky.app/profile/superbugs.bsky.social/post/3lsq7wq2p5s22	29/06/2025	9 likes, 5 reposts
Superbugs	https://bsky.app/profile/superbugs.bsky.social/post/3lt5bbgfibk2i	04/07/2025	6 likes, 2 reposts
Superbugs	https://bsky.app/profile/superbugs.bsky.social/post/3ltdfgo6cjc26	07/07/2025	8 likes, 5 reposts
Superbugs	https://bsky.app/profile/superbugs.bsky.social/post/3ltf2r7dhrc2a	07/07/2025	4 likes, 3 reposts
Superbugs	https://bsky.app/profile/superbugs.bsky.social/post/3ltelhha4g22j	07/07/2025	5 likes, 4 reposts
Superbugs	https://bsky.app/profile/superbugs.bsky.social/post/3lthn6zpggc24	08/07/2025	6 likes, 5 reposts
Superbugs	https://bsky.app/profile/superbugs.bsky.social/post/3lthnffkcts24	08/07/2025	5 likes, 3 reposts
Superbugs	https://bsky.app/profile/superbugs.bsky.social/post/3ltkg7o2d722d	09/07/2025	7 likes, 3 reposts
Superbugs	https://bsky.app/profile/superbugs.bsky.social/post/3ltkdmiectk2d	09/07/2025	6 likes, 2 reposts
Superbugs	https://bsky.app/profile/superbugs.bsky.social/post/3ltoajmkzoc2g	11/07/2025	5 likes, 3 reposts
Superbugs	https://bsky.app/profile/superbugs.bsky.social/post/3ltpdg23u5k2y	11/07/2025	4 likes, 2 reposts
Superbugs	https://bsky.app/profile/superbugs.bsky.social/post/3ltpdqeeu5s2y	11/07/2025	15 likes, 7 reposts
Superbugs	https://bsky.app/profile/superbugs.bsky.social/post/3ltrxe5jcqc2u	12/07/2025	2 likes, 1 repost
Superbugs	https://bsky.app/profile/superbugs.bsky.social/post/3lts5ouybq227	12/07/2025	6 likes, 4 reposts
Superbugs	https://bsky.app/profile/superbugs.bsky.social/post/3ltuomibl722u	13/07/2025	5 likes, 2 reposts
Superbugs	https://bsky.app/profile/superbugs.bsky.social/post/3ltuktd2cwc2n	13/07/2025	4 likes, 2 reposts
Superbugs	https://bsky.app/profile/superbugs.bsky.social/post/3ltuoemx4sc2u	13/07/2025	5 likes, 4 reposts
Superbugs	https://bsky.app/profile/superbugs.bsky.social/post/3ltwvqltgjk2x	14/07/2025	4 likes, 1 repost
Superbugs	https://bsky.app/profile/superbugs.bsky.social/post/3ltx4nht4ls2x	14/07/2025	8 likes, 7 repost
Superbugs	https://bsky.app/profile/superbugs.bsky.social/post/3ltzbbndcmc2t	15/07/2025	3 likes, 2 reposts
Superbugs	https://bsky.app/profile/superbugs.bsky.social/post/3ltzpd45kgc2f	15/07/2025	15 likes, 8 reposts
Superbugs	https://bsky.app/profile/superbugs.bsky.social/post/3lu2vet252k2t	16/07/2025	11 likes, 4 reposts
Superbugs	https://bsky.app/profile/superbugs.bsky.social/post/3lu3xacds5c2l	16/07/2025	11 likes, 5 reposts

Superbugs	https://bsky.app/profile/superbugs.bsky.social/post/3lu3woiag2k2l	16/07/2025	5 likes, 1 repost
Superbugs	https://bsky.app/profile/superbugs.bsky.social/post/3lu3wjdy7ss2l	16/07/2025	5 likes, 1 repost
Superbugs	https://bsky.app/profile/superbugs.bsky.social/post/3lu37yri2n22t	16/07/2025	2 likes, 2 reposts
Superbugs	https://bsky.app/profile/superbugs.bsky.social/post/3lu6w62zdrs2f	17/07/2025	3 likes, 2 reposts
Superbugs	https://bsky.app/profile/superbugs.bsky.social/post/3lubjklswds2y	18/07/2025	3 likes, 2 reposts
Superbugs	https://bsky.app/profile/superbugs.bsky.social/post/3lucefqycas2i	19/07/2025	12 likes, 5 reposts
Superbugs	https://bsky.app/profile/superbugs.bsky.social/post/3luhhkced6k2z	21/07/2025	5 likes, 7 reposts
Superbugs	https://bsky.app/profile/superbugs.bsky.social/post/3luk6hy3msc2a	22/07/2025	7 likes, 6 reposts
Victor Nizet	https://bsky.app/profile/nizet.bsky.social/post/3lulead4a5c2e	22/07/2025	26 likes, 2 reposts
Superbugs	https://bsky.app/profile/superbugs.bsky.social/post/3luntcvkjtc2k	23/07/2025	5 likes, 2 reposts
Superbugs	https://bsky.app/profile/superbugs.bsky.social/post/3lv4lwjh5rc2r	29/07/2025	5 likes, 3 reposts
Superbugs	https://bsky.app/profile/superbugs.bsky.social/post/3lv4lm7l4sc2r	29/07/2025	7 likes, 3 reposts
Superbugs	https://bsky.app/profile/superbugs.bsky.social/post/3lv6cb2vdbk2o	30/07/2025	25 likes, 7 reposts
Superbugs	https://bsky.app/profile/superbugs.bsky.social/post/3lv75tjn4vs2f	30/07/2025	3 likes, 2 reposts
Superbugs	https://bsky.app/profile/superbugs.bsky.social/post/3lvaq2227ts2w	31/07/2025	22 likes, 10 reposts
Superbugs	https://bsky.app/profile/superbugs.bsky.social/post/3lvb5kjdod22h	31/07/2025	9 likes, 3 reposts
Superbugs	https://bsky.app/profile/superbugs.bsky.social/post/3lve4skapc22z	01/08/2025	5 likes, 3 reposts
Superbugs	https://bsky.app/profile/superbugs.bsky.social/post/3lve4oivk622z	01/08/2025	3 likes, 2 reposts
Superbugs	https://bsky.app/profile/superbugs.bsky.social/post/3lvd4nsvias2z	01/08/2025	13 likes, 3 reposts
Superbugs	https://bsky.app/profile/superbugs.bsky.social/post/3lvd4a5jpyk2g	01/08/2025	8 likes, 2 reposts
Superbugs	https://bsky.app/profile/superbugs.bsky.social/post/3lvgtymybqs2p	02/08/2025	5 likes, 3 reposts
Superbugs	https://bsky.app/profile/superbugs.bsky.social/post/3lvjfpzehsc2g	03/08/2025	3 likes, 2 reposts
Superbugs	https://bsky.app/profile/superbugs.bsky.social/post/3lvjfwxp6p22g	03/08/2025	13 likes, 5 reposts
Superbugs	https://bsky.app/profile/superbugs.bsky.social/post/3lvjellduuk2g	03/08/2025	4 likes, 3 reposts
Superbugs	https://bsky.app/profile/superbugs.bsky.social/post/3lvjejrs4ds2g	03/08/2025	9 likes, 3 reposts
Superbugs	https://bsky.app/profile/superbugs.bsky.social/post/3lvlx7chc7s2a	04/08/2025	9 likes, 2 reposts
Superbugs	https://bsky.app/profile/superbugs.bsky.social/post/3lvnjca6gok2o	05/08/2025	5 likes, 2 reposts
Superbugs	https://bsky.app/profile/superbugs.bsky.social/post/3lvnc27zvfc2x	05/08/2025	21 likes, 7 reposts
Superbugs	https://bsky.app/profile/superbugs.bsky.social/post/3lvof75mlr22v	05/08/2025	4 likes, 2 reposts
Superbugs	https://bsky.app/profile/superbugs.bsky.social/post/3lw262vdkfc2m	10/08/2025	13 likes, 10 reposts
Superbugs	https://bsky.app/profile/superbugs.bsky.social/post/3lwgin2twis2b	15/08/2025	8 likes, 2 reposts

Supplementary Table 3. Qualitative feedback from pop-up shop visitors as extracted from the post-event questionnaires.

**Free text answers to the question *“Please tell us what you liked most about ‘Superbugs’?”***
The variety of things to do.
A lot
Ball pit
Collecting 6 stamps
Coloring
Engaging activity with the kids
Engaging for children
Engaging staff
Enjoyed and liked looking at the bugs
Everyone was willing to help
Everything
Free, staff very friendly
Fun activities
Fun and free activities for learning
Good treasure hunt and my son loved the VR experience
great interactive experience
Growing your own
Handwash challenge
Horror show
Horror show with physical evidence
How interactive it is, the staff were amazing and we learnt so much. All of this and it's free!
how interactive it was
how interactive the learning was
Informative and fun
Informative. Able to ask questions to real people with knowledge and hands on experience
Interactive
Interactive
it increased my awareness of bacteria around me and gave me invaluable knowledge on infections, viruses and
It was free
It was just like really cool
It was very informative and interactive
Lego
Liked the beanbag throwing
Liked the spike ball pit find
Lots of different activities, kind and patient staff, prizes to help engage the kids!
Lots of variety. Think it is pitched at slightly older children but still learnt and nice to see not dumbing down.
Loved every bit of it- best one was antibiotics killing bugs (bean bag game)
Lovely staff
Microscope
Microscope
My kid says coloring the bugs and the stamps quest
Raising the awareness about the benefits of vaccines and basic hygiene.
Really interactive
Staff and innovative ways to get children to learn

Staff very informative
Staff were great, very friendly to kids
Staff were lovely. My kids loved the experience. Well done all. Fantastic initiative.
Super friendly staff and lots of fun games
The activities and VR headset
The antibiotics resistance game
The assistants answering questions rather than just reading ourselves
The bean bags throwing. It was detailed and fun.
The Children were very engaged
The engaging activities especially grow your own microbe
The grow your own microbes
The hand-wash and vr and the horror show
the interactive aspect
The people working there
The treasure hunt
The treasure hunt and the horror show
the visual pictures
Thr activity
Treasure hunt
Treasure hunt
Very engaging and educational – great staff
Virtual experience
Virtual lab
vr
VR
VR experience
We'll run and fun !!!
X
**Free text answers to the question *"How could we improve 'Superbugs' events in future?"***
A few more people manning the different activities, some simply analogies would have been helpful to explain
Aln support
Audio explanation on every activity
Buy giant microbes and books
Cooler. Very very hot.
For a youngster it was pitched perfectly
Get that the bugs need their proper scientific name but maybe give them shorter names for treasure hunt.
I don't think you could – maybe a fact sheet that summarises key points that are easy to remember
I don't see how!
It was good
It wasn't clear if some activities were 'open' – eg we weren't sure whether we could use the microscopes as we
It's great as it was
M/A
Make it more kid friendly
More about antibiotics
More activities
more adult based info
more advertisement
More advertising

More assistants
More funding would ramp everything up a bit!
More germs on the treasure hunt
more guidance
More interaction and enthusiasm from students / staff
More microbes
More prizes
More staff although they tried very hard! :-)
More vr
My son found the microbial horror show scary and possibly a bit beyond him in terms of the amount of
N/a
N/a
N/a
No
No newd
Not sure, it's pretty cool! Maybe easier language for little ones, but I understand it's not the target age
nothing
nothing
nothing
Nothing! It was amazing!
Nothing, it was great, my 5yo couldn't absorb *everything*, but he did learn and enjoyed it very much.
put some music on in background
Roll it out to other cities
Room was very warm so water for children
Some games are a bit hard.
The Horror show
To suit a younger audience

Supplementary Table 4. Demographics of regular pop-up shop visitors completing the long-term impact survey (*n*=21).

**UK postcode district**	% of respondents
CF3	4.8
CF5	4.8
CF11	4.8
CF14	9.5
CF23	9.5
CF24	9.5
CF35	4.8
CF63	4.8
CF64	9.5
CF72	19.0
NP11	4.8
NP44	4.8
SA65	4.8
TA2	4.8
**Number of children per visitor**	
0	4.8
1	47.6
2	33.3
3	4.8
4	9.5
**Age of children (years)**	
<3	2.9
3–6	51.4
7–10	31.4
11–14	14.3
>14	0
**Distance of childrens' school from event**	
5 km and less	23.8
6–10 km	33.3
11–20 km	0
21–30 km	23.8
31–40 km	14.3
>40 km	4.8

Supplementary Table 5. Qualitative feedback from regular pop-up shop visitors as extracted from the long-term impact survey.

**Free text answers to the question *"In your opinion, which content or activity in the pop-up shop worked best?"***
Interactive activities worked really well with my daughter. The visual approach was fantatic
VR headset, microscope slides, having a sheet to fill out kept him engaged.
Microscopes
Hands on events and "doing" learning
VR
All activities
The interactive bits
The swabs for germs
for the children – the agar plates and the ball bit
activities and trails
the hand gel and the ultra violet light
The handwashing station – my son is doing a surprisingly good job!
For our basic understanding/ age of the children the hand washing machine/ demonstration made more sense
The treasure hunt
The microscope
Killing bugs with antibiotics (hitting tins), microscopic view of bugs
growing bacteria in petro dishes, vr
Horror Tunnel , wash hands, throwing the germs, 3D video
Practical activities such as incubating swabs and seeing bacteria growth
I genuinely liked all of them
**Free text answers to the question *"Which other scientific topic would you like to see presented in a public pop-up shop?"***
Any
Anything, my child loves all things science.
STEM for girls
We loved this one so any with a practical element
Health
Human organs/ in healthy and diseased
The environment
space, ocean and pollution in oceans seas
No preference
Healthy eating e.g. gut health, sugar
**Free text answers to the question *"Please list essential criteria of future science pop-up shops."***
Free entry
Free/cheap, accessible location, age appropriate activities to keep young children engaged.
Practical elements
free entry and fun for children
Engaging
Health lung and COPD, the heart and CVD, ECG
Child friendly
easy access, free access
Accessible, free fun
Accessible location, free or low cost

Supplementary Table 6. Demographics of pop-up shop helpers completing the post-event survey (*n*=33).

	% of respondents
**Age in years**	
<21	15.2
21–30	54.5
31–40	9.1
41–50	15.2
51–60	3.0
>60	3.0
**Career stage / Job title**	
A level pupil or equivalent	9.1
Undergraduate student	27.3
Postgraduate student (MSc, PhD or equivalent)	36.4
Postdoctoral researcher or equivalent	6.1
Lecturer / Senior Lecturer	6.1
Assoc. Prof. / Professor	3.0
Professional Services	9.1
Other	3.0
**Affiliation**	
Cardiff University	54.8
Cardiff Metropolitan University	25.8
Swansea University	19.4
Other	3.2
**Scientific discipline**	
Biomedical Sciences	66.7
Medicine / Healthcare Sciences	15.2
Biosciences / Natural Sciences	15.2
Psychology / Social Care	0
Mathematics / Physics	0
Other	12.1
**Prior experience in public engagement**	
Never participated	33.3
Once per year	39.4
Once every 6 months	18.2
Once a month	6.1
Greater than once a month	3.0
**Participation in Superbugs**	
Delivery of activities to general public	84.8
Delivery of activities for school visits	51.5
Planning and development of content/activities	27.3
Preparation of shop location	21.2
Evaluation and data collection	12.1

Supplementary Table 7. Presentations by the Superbugs team showcasing learnings from the pop-up shop to diverse audiences. Numbers of delegates/attendees and festival visitors were obtained from the organisers (personal communication).

Organiser	Event	Main audience	Delegates/ attendees	Location, date	Type of presentation
Hendredenny Park Primary School	Superbugs workshop at local primary school (Year 5 pupils)	Public	35	Caerphilly, 9 & 13 Oct 2025	Interactive exhibition
Swansea University	Swansea Science Festival 2025, National Waterfront Museum	Public	4,886	Swansea, 25–26 Oct 2025	Interactive exhibition
Cardiff University	One-day conference: “Engaging with Purpose: Global-Civic Collaborations for Future Generations”	Professional	250	Cardiff, 5 Nov 2025	Invited talk
British Academy	One-day seminar: “Communicating for Change: Advancing Just Transitions for the Equitable and Sustainable Mitigation of Antimicrobial Resistance”	Professional	46	London, 7 Nov 2025	Invited talk
Cardiff University	One-day conference: “School of Medicine Patient and Public Involvement and Engagement Showcase Event”	Mixed	152	Cardiff, 18 Nov 2025	Invited talk and poster
Cardiff University	Annual Infection & Immunity Meeting 2025	Professional	226	Cardiff, 27 Nov 2025	Oral and abstract
Cardiff University	Science in Health Public Lecture Series 2025/2026	Public	97 live; 106 views on YouTube*	Cardiff, 27 Nov 2025	Public lecture (virtual)
British Society for Immunology	Annual Congress 2025	Professional	1,589	Liverpool, 1–4 Dec 2025	Poster and abstract
UK Dementia Research Institute	Institute seminar (Cardiff campus)	Professional	approx. 25	Cardiff, 26 Jan 2026	Invited talk

* as of 12 February 2026. Recordings are available at *www.youtube.com/watch?v=YCSbL9DZTh0* and *www.youtube.com/watch?v=ASwVGe9N-9M*.

SUPPLEMENTARY FIGURES

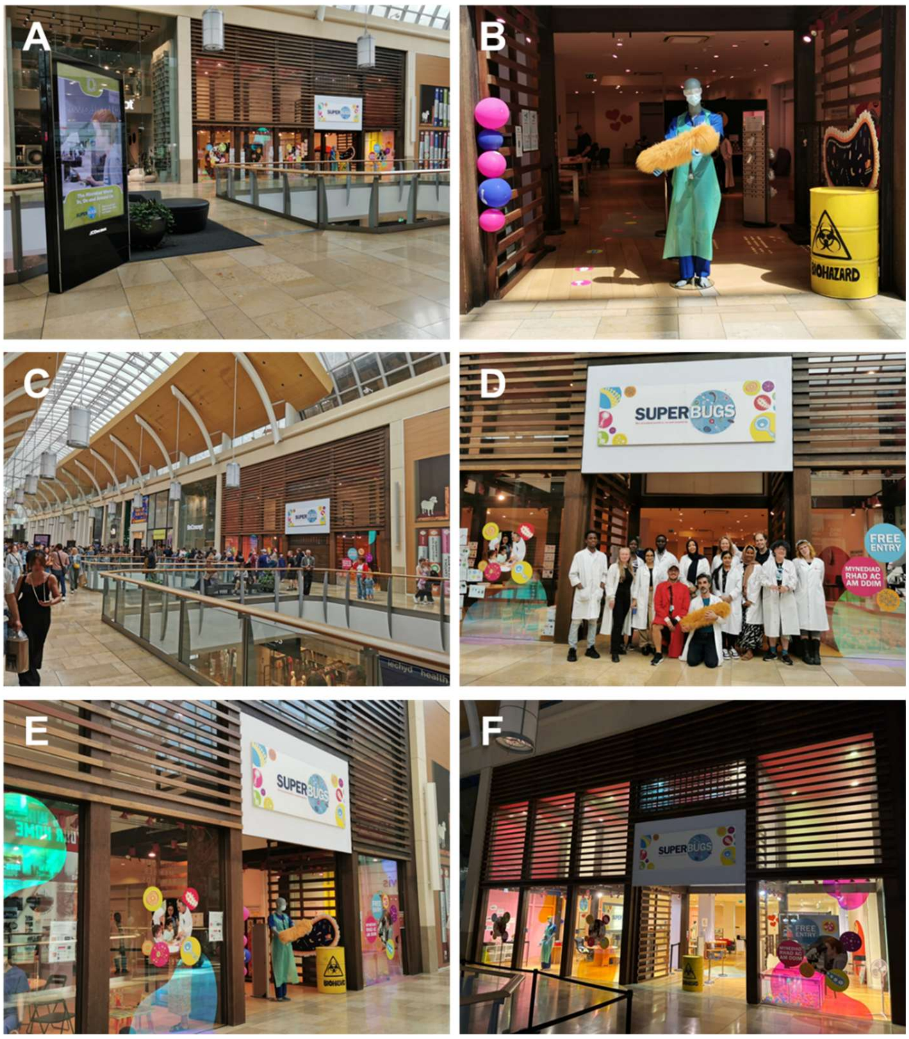

Supplementary Figure 1. Pop-up shop front. ***(A)*** View across the shopping centre with digital advert in the foreground; ***(B)*** shop entrance with a mannequin holding a giant *E. coli* 'superbug'; ***(C)*** footfall two days before the pop-up shop opened (the weekend the band Oasis played two reunion concerts in Cardiff), with people queueing outside the 'Oasis' merchandise store located directly opposite the Superbugs pop-up shop; ***(D)*** Superbugs team members posing on the last day of the event; ***(E)*** close-up of the shop windows during daytime and ***(F)*** in the evening.

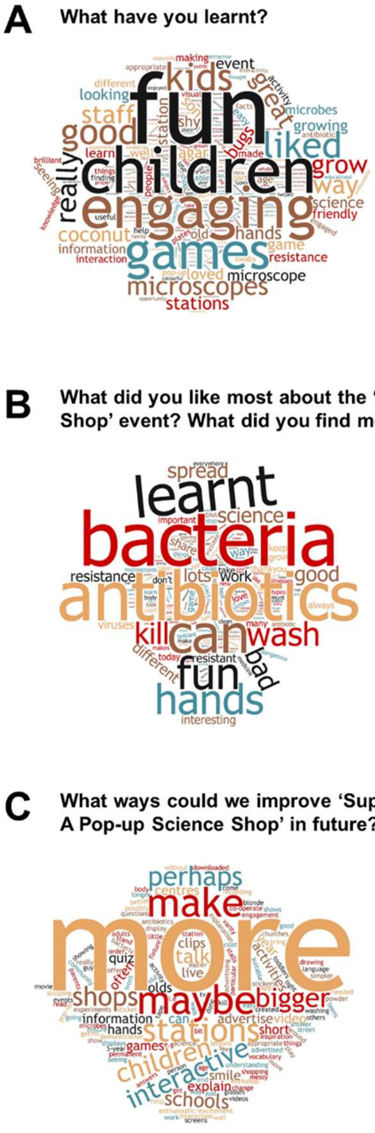

Supplementary Figure 2. Word clouds summarising feedback received by visitors of the 2019 event. ***(A)*** Summary of feedback from *n*=116 post-it notes on a 'bacterial thought tree'; ***(B)*** summary of *n*=202 responses to the question shown from a questionnaire completed by visitors of the 2019 event; ***(C)*** summary of *n*=53 responses to the question shown from a questionnaire completed by visitors of the 2019 event. The 2019 event was described in detail in Tyrrell et al., 2022.

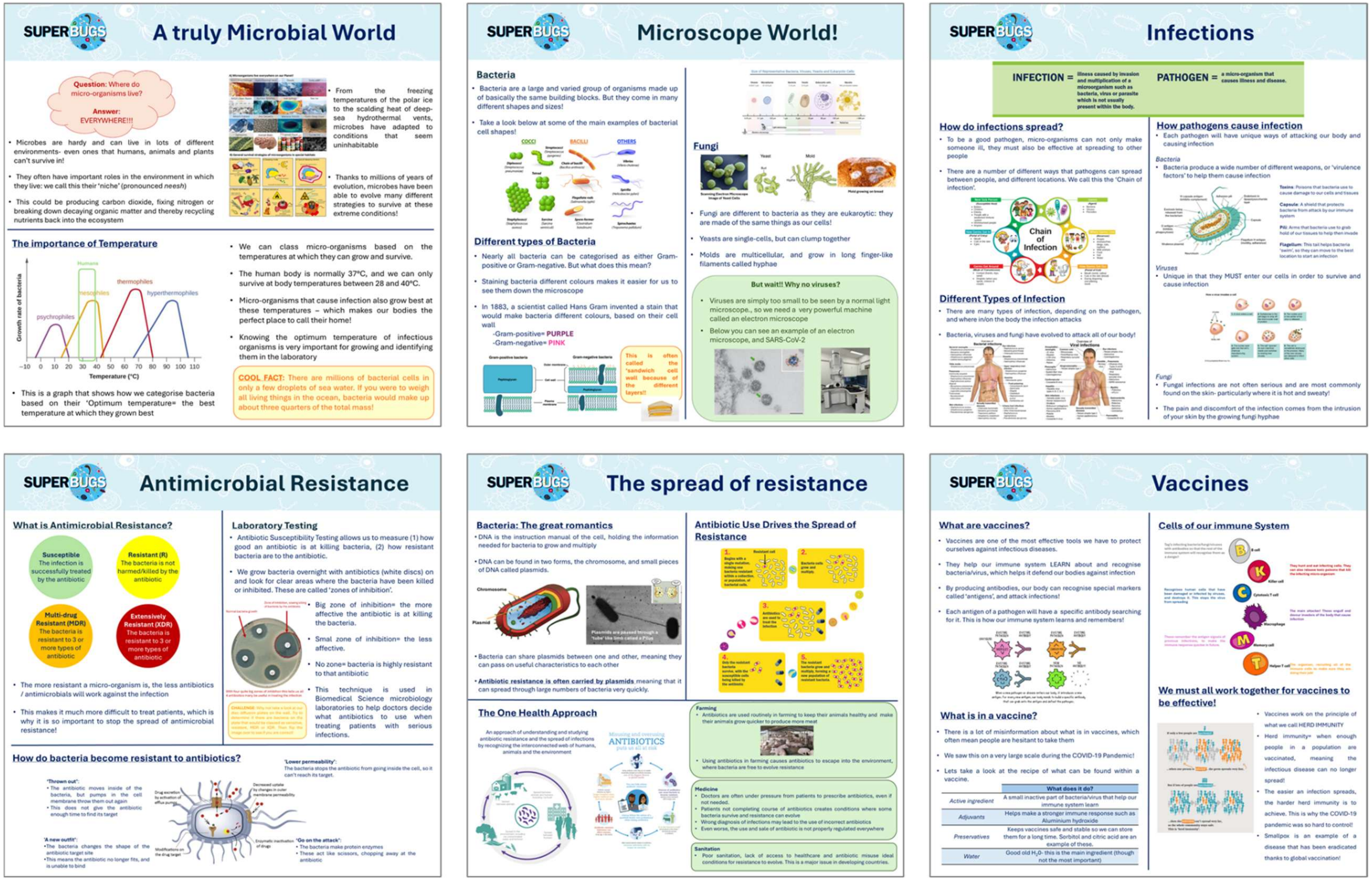

Supplementary Figure 3. Foamex infoboards. Large-scale professionally printed infoboards explaining the scientific background for major activities in the pop-up shop. All infoboards were provided both in English and in Welsh (not shown here).

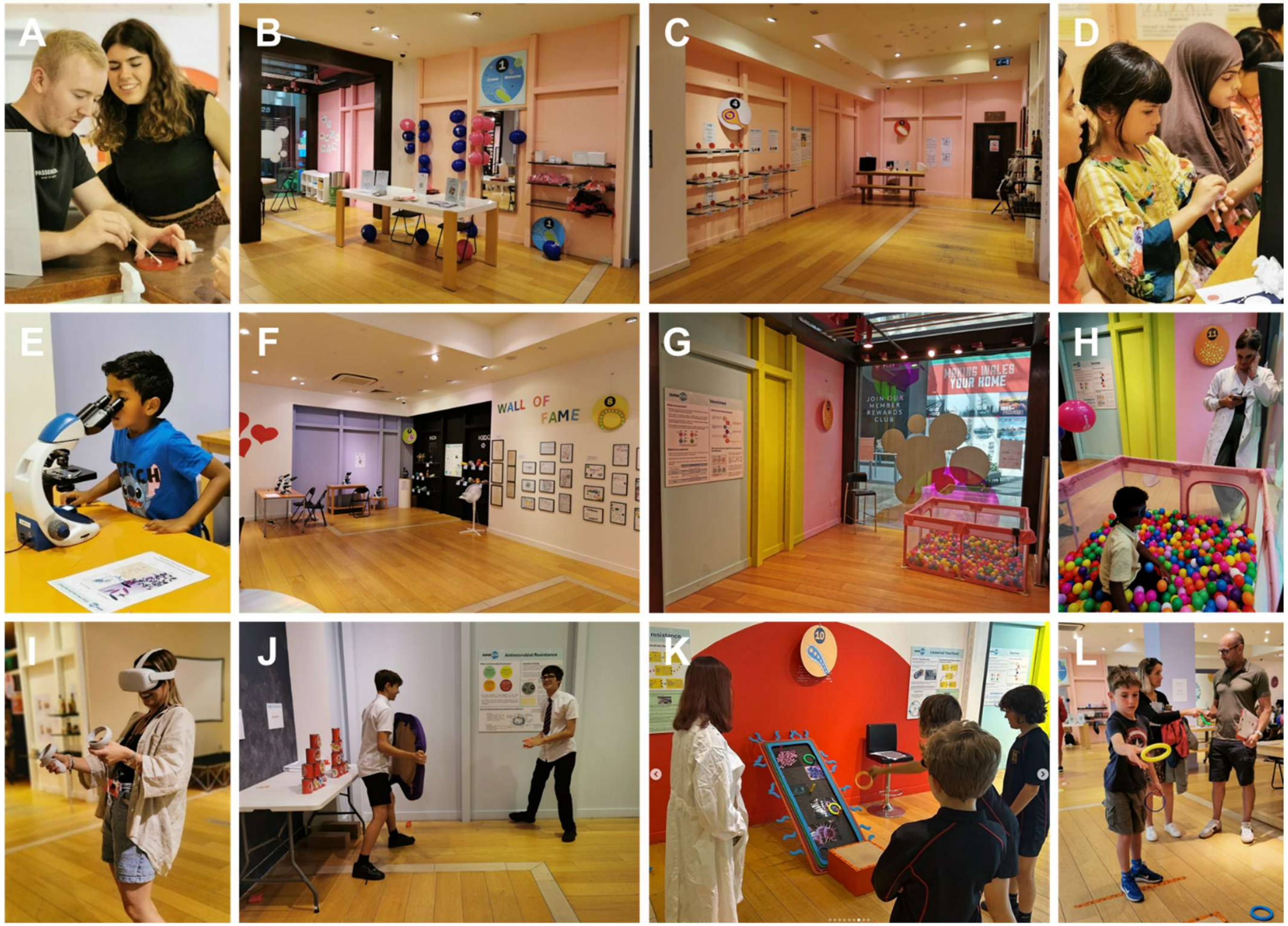

Supplementary Figure 4. Main shop activities for the sticker challenge. ***(A)*** Grow your own microbes; ***(B)*** welcome desk with activity sheets and prizes; ***(C, D)*** agar plate display and handwashing activity; ***(E, F)*** microscope station and microbial shapes; ***(G, H)*** vaccine ball pit; ***(I)*** VR headsets; ***(J)*** AMR tin can alley; ***(K, L)*** plasmid ring toss.

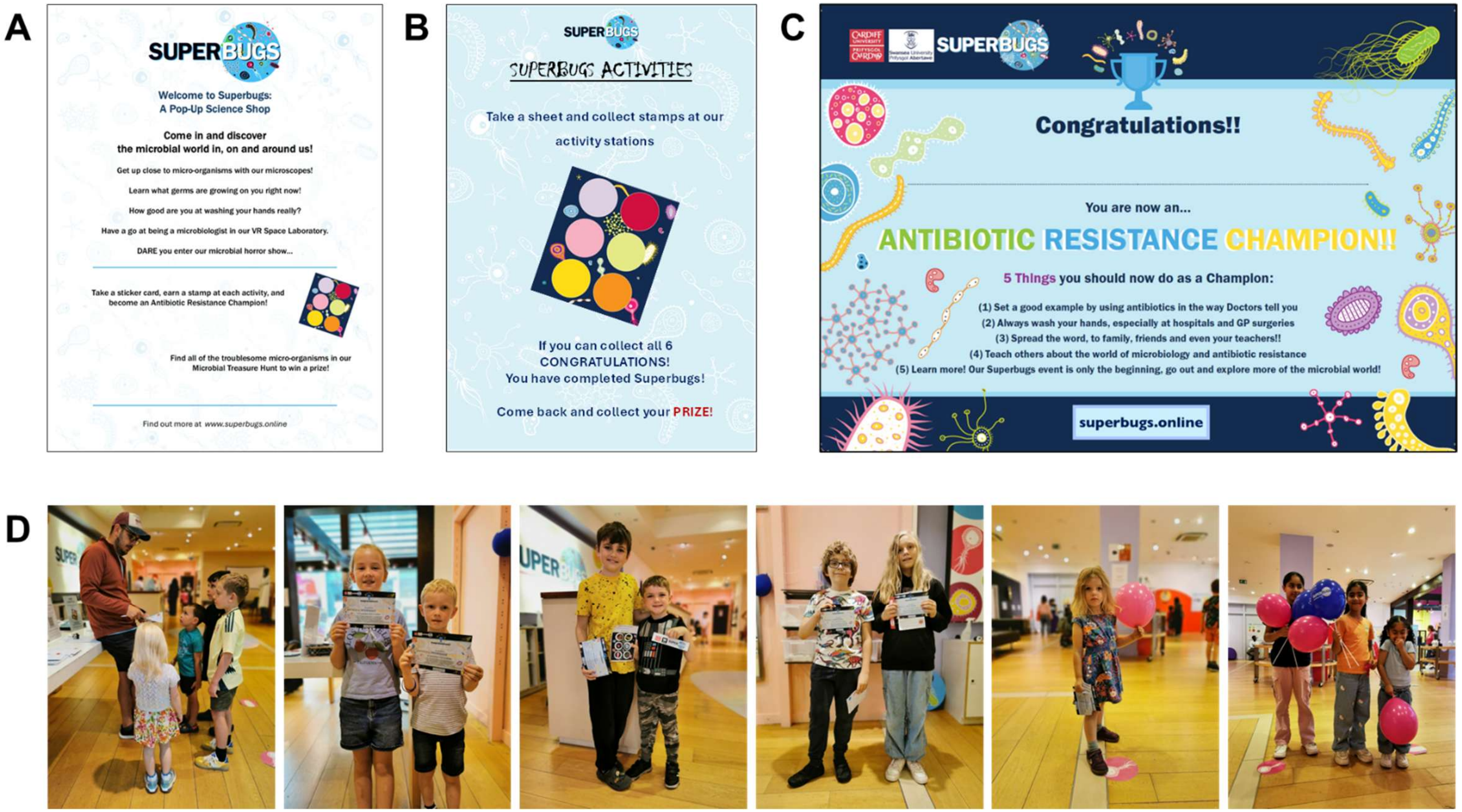

Supplementary Figure 5. Sticker challenge. ***(A)*** Welcome sheet; ***(B)*** sticker challenge instructions as provided at the welcome desk; ***(C)*** Antibiotic Resistance Champion certificate; ***(D)*** visitors who successfully completed the sticker challenge. The activity including instructions and certificate was provided both in English and Welsh (not shown here).

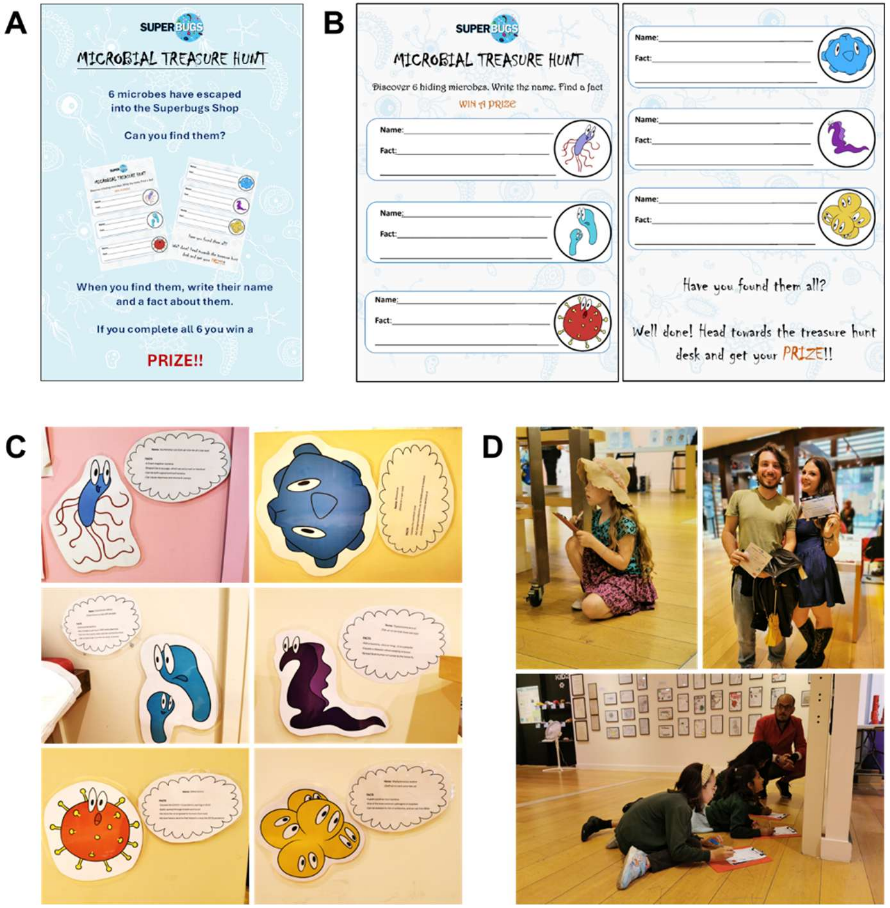

Supplementary Figure 6. Treasure Hunt. ***(A)*** Instructions as provided at the welcome desk; ***(B)*** treasure hunt sheets; ***(C)*** 'nasty germs' that escaped into the shop: *E. coli*, *Clostridioides difficile*, SARS-CoV-2, rhinovirus, *Trypanosoma brucei* and *Staphylococcus aureus*; ***(D)*** visitors engaged in the activity. The treasure hunt activity was provided both in English and Welsh (not shown here).

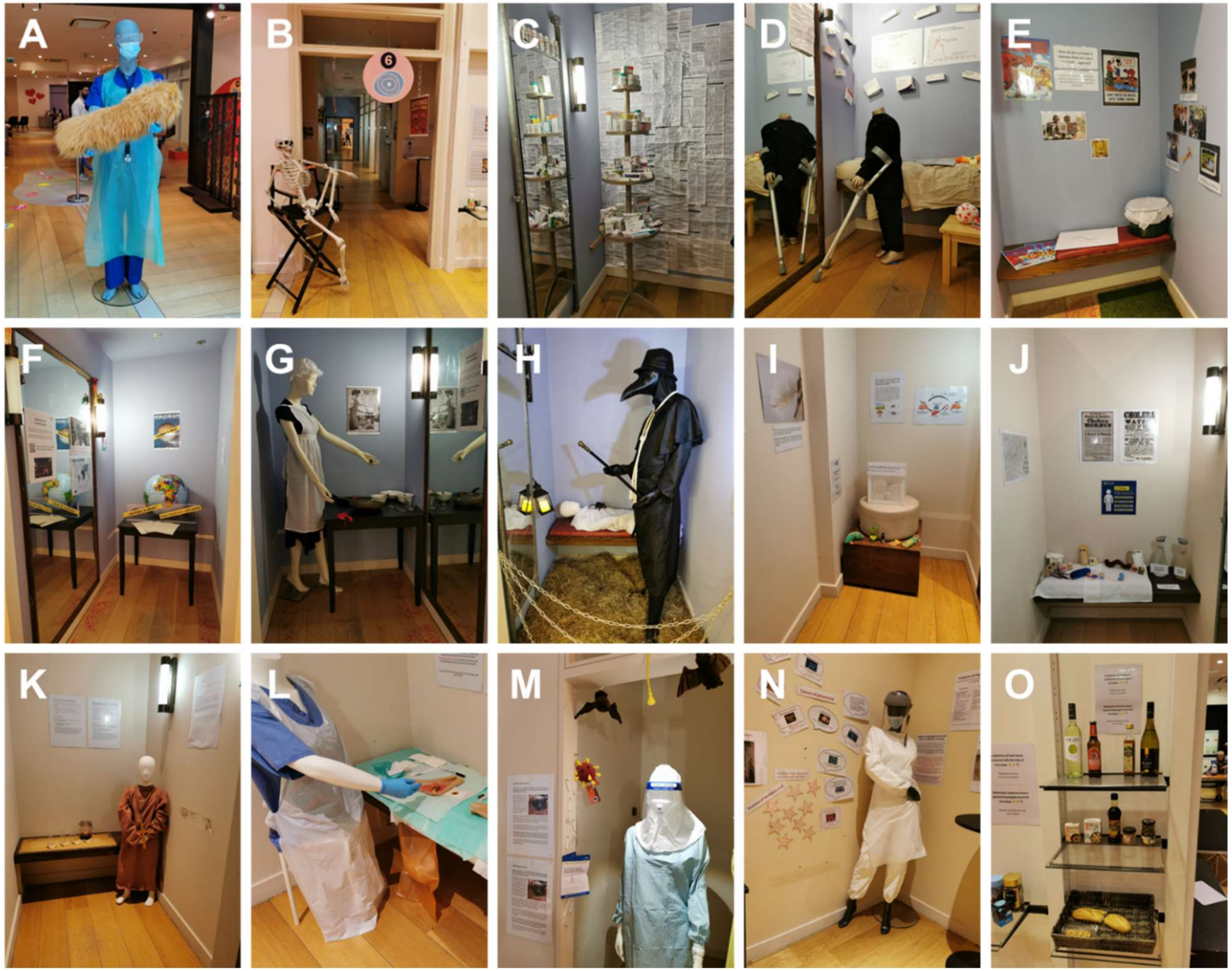

Supplementary Figure 7. Superbugs Horror Show displays. ***(A)*** Shop entrance; ***(B)*** horror show entrance; ***(C)*** antimicrobial resistance; ***(D)*** measles and polio; ***(E)*** parasitic worms; ***(F)*** smallpox; ***(G)*** typhoid; ***(H)*** Black Death; ***(I)*** malaria; ***(J)*** cholera; ***(K)*** leprosy; ***(L)*** wound infections; ***(M)*** COVID-19 and Ebola; ***(N)*** infection diagnosis; ***(O)*** friendly germs.

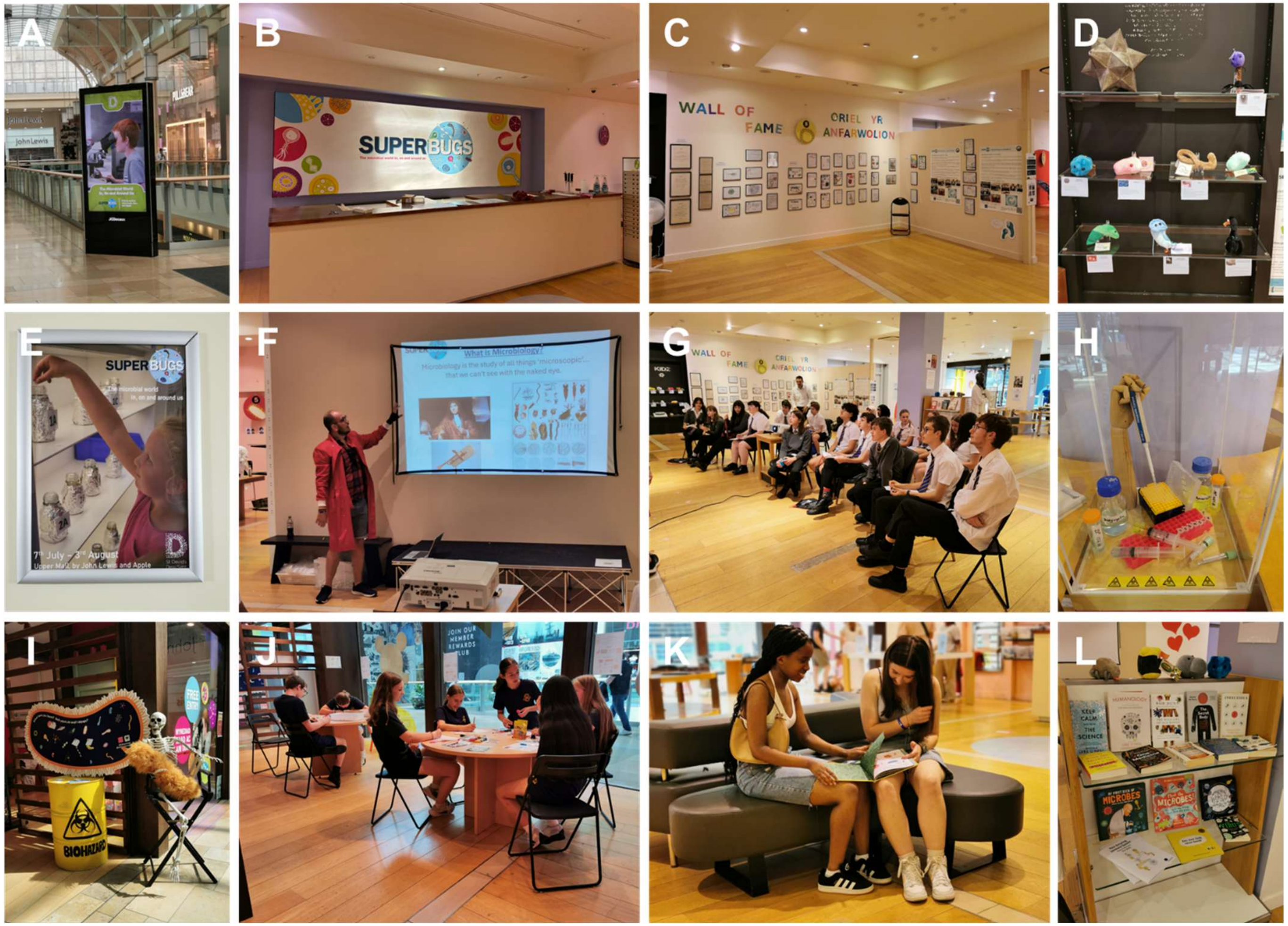

Supplementary Figure 8. Additional shop features. ***(A)*** Large-scale adverts on digital screens; ***(B)*** Superbugs banner behind the swabbing station ; ***(C)*** gallery with school artwork and poems; ***(D)*** microbial shapes display; ***(E)*** printed posters displayed in elevators and stairwells; ***(F*, *G)*** live stage for presentations and quizzes; ***(H)*** laboratory items display; ***(I)*** bacterial thought-tree for feedback; ***(J)*** arts and crafts corner; ***(K)*** reading corner and sitting area; ***(L)*** bookshelf with popular science books.

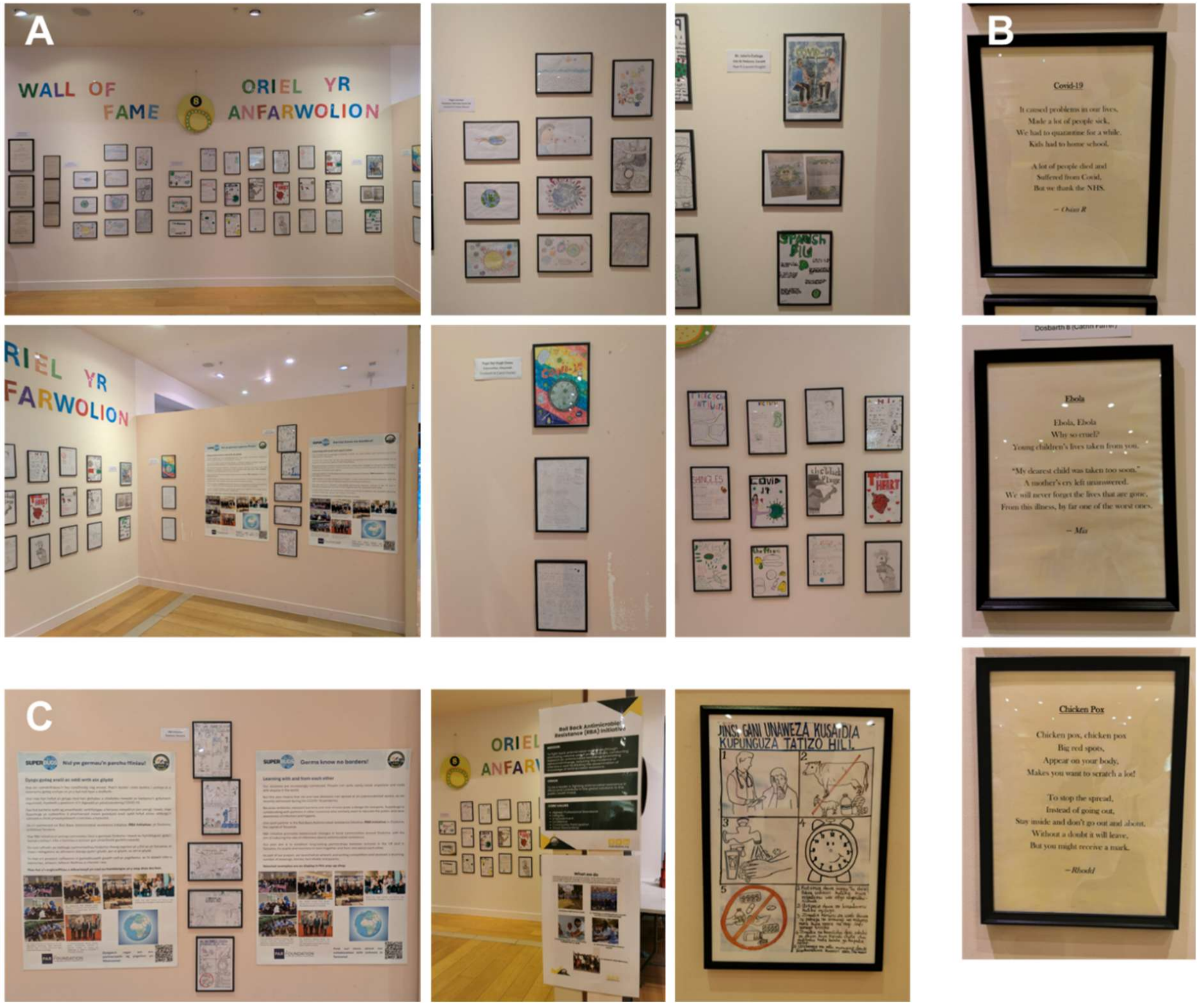

Supplementary Figure 9. School art exhibition. Gallery of framed artwork *(A)* and written work *(B)* by pupils from various secondary schools in Wales; *(C)* overview of Superbugs collaboration with RBA Initiative and examples of artwork by pupils in Dodoma, Tanzania.

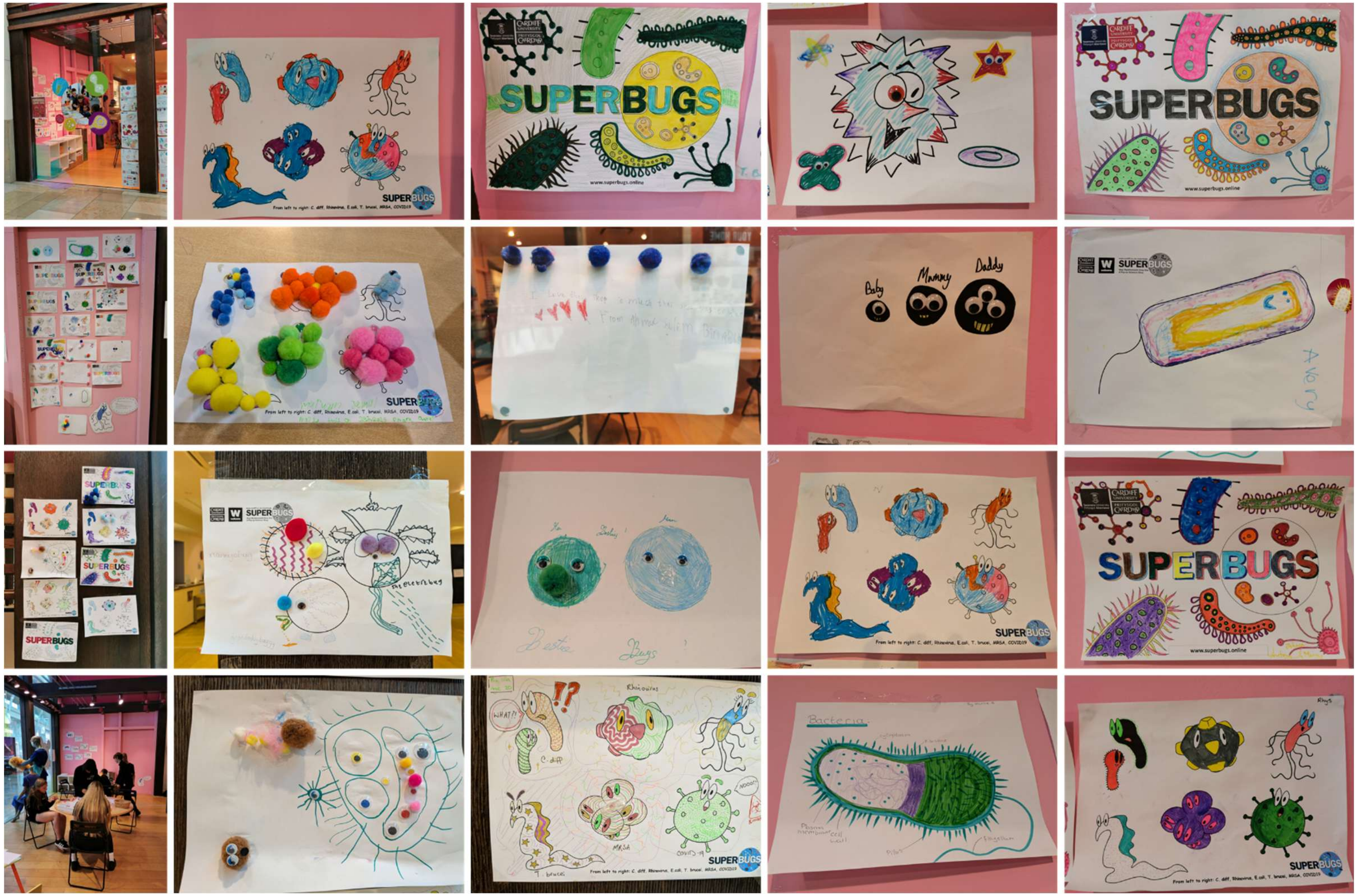

Supplementary Figure 10. Arts and crafts corner. Representative examples of drawings created by visitors of the pop-up shop.

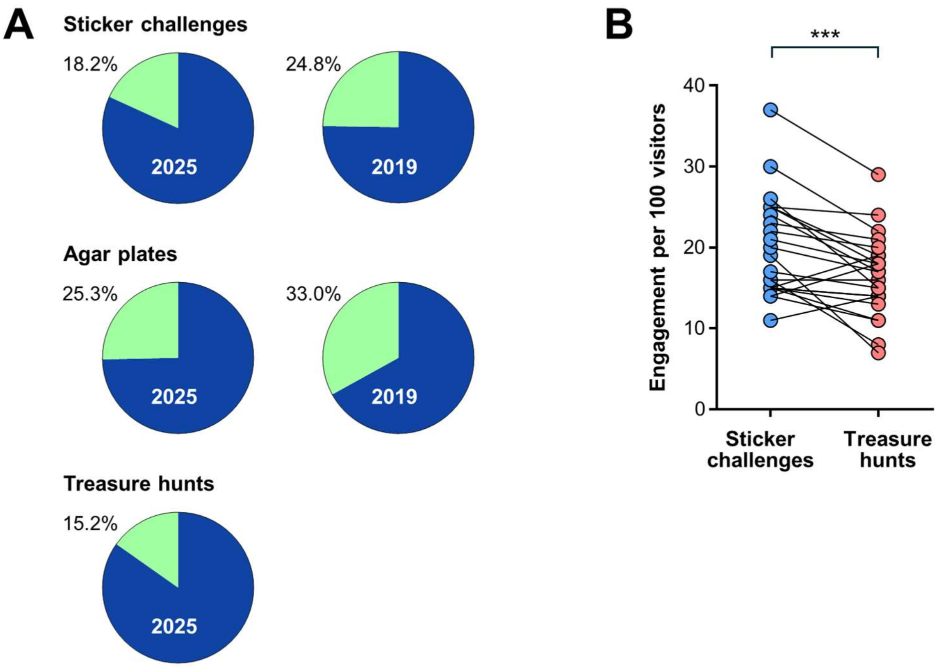

Supplementary Figure 11. Visitor engagement rates. (***A***) Relative proportion of sticker challenges, body swabs and treasure hunts completed (in green) by visitors of the 2025 (left) and 2019 (right) pop-up shops. (***B***) Comparison of engagement rates for the sticker challenge and treasure hunt activities; each data point represents a different day. Statistical significance was assessed using a paired t-tests: ***, $p<0.001$.

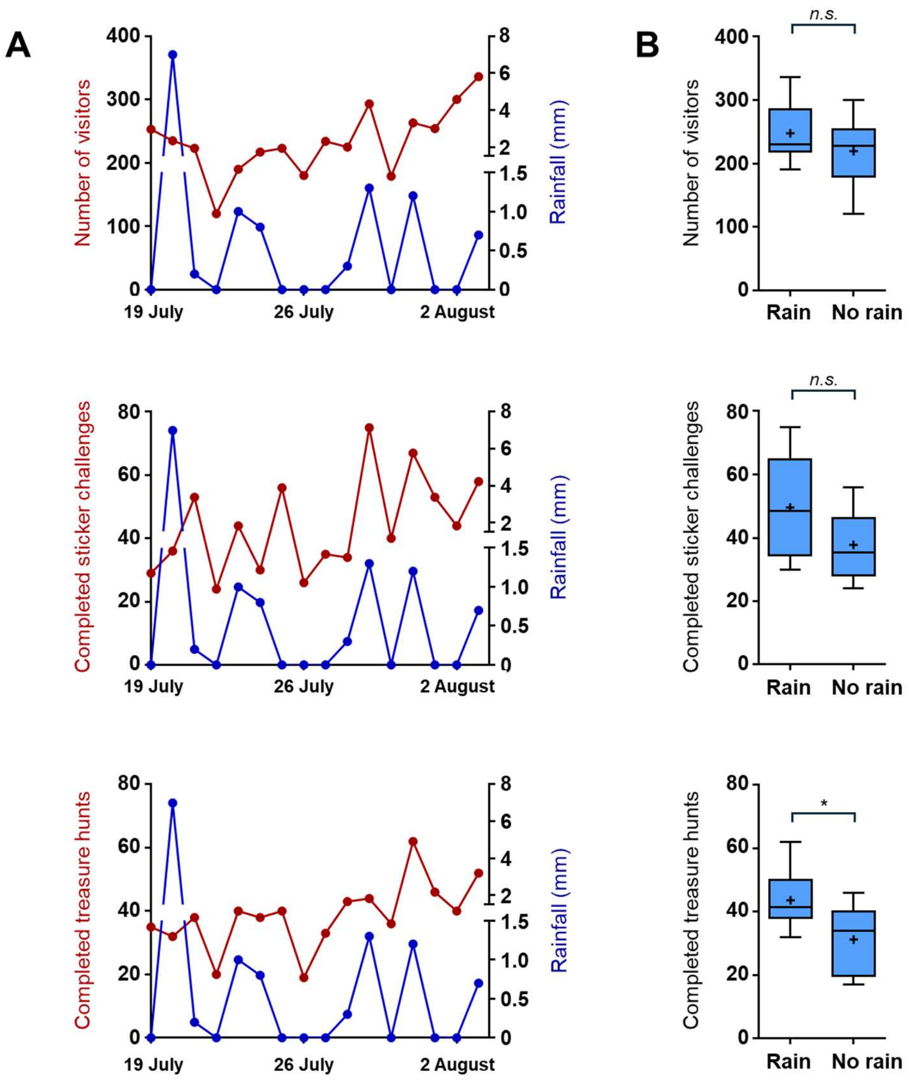

Supplementary Figure 12. Correlation between daily pop-up shop activities and the weather. (***A***) Daily number of visitors, completed sticker challenges and completed treasure hunts between 19 July and 3 August 2025, together with the daily precipitation between 9:00 and 17:00 on each day. (***B***) Average daily number of visitors, completed sticker challenges and completed treasure hunts on public days (12/13 July and again between 19 July and 3 August 2025) with and without rainfall between 9:00 and 17:00. Tukey whisker plots show upper and lower quartiles as boxes above and below the median line, and whiskers representing the 1.5 interquartile range; the mean is depicted by the plus symbol. Statistical significance was assessed using Mann-Whitney tests: *, $p<0.05$; *n.s.*, not significant.

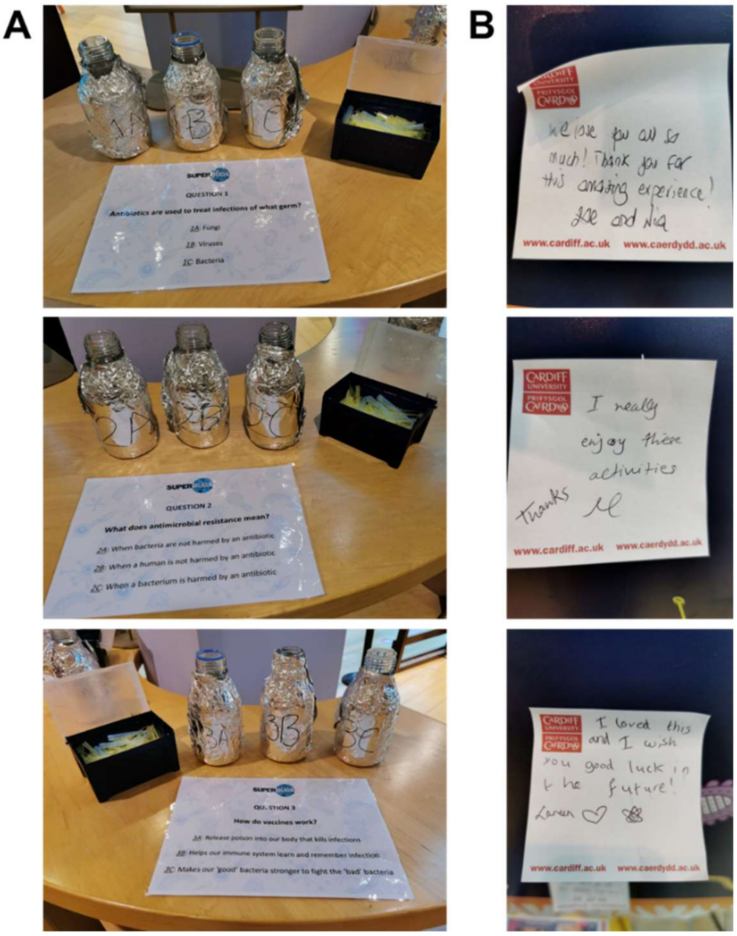

Supplementary Figure 13. Quantitative and qualitative impact evaluation. (***A***) Set of benchmarking questions at the shop entrance; an identical set of questions was also used at the shop exit (not shown). Visitors were asked to put a plastic pipette tip into the glass bottle representing the right answer; bottles were wrapped in foil to conceal the content and ensure responses would not be influenced by previous visitors. Bottles were emptied each evening and pipette tips were counted manually. (***B***) Examples of post-it notes left by visitors at the bacterial ‘thought tree’ at the shop exit.

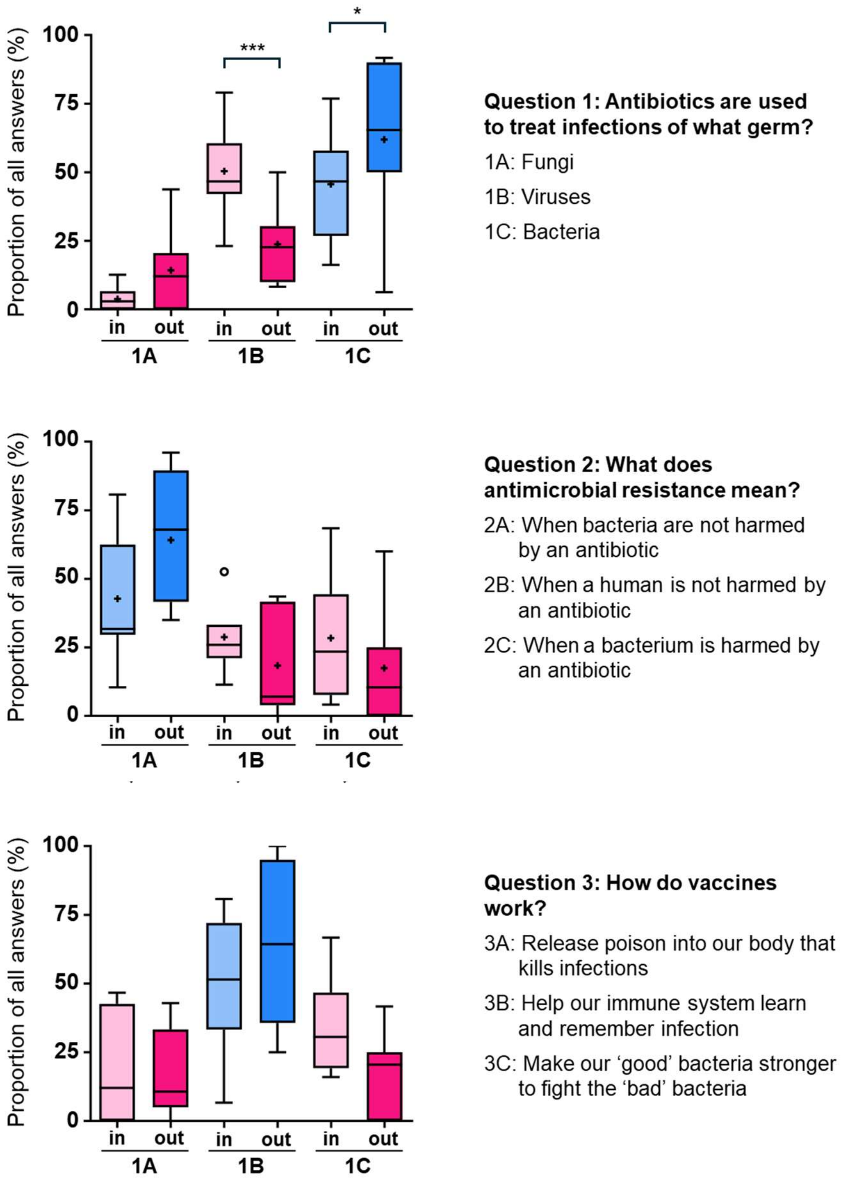

Supplementary Figure 14. Assessment of learning outcomes for a total of seven school visits. At each visit, pupils were asked the three questions shown at the beginning (in; n= 247, 209 and 216, respectively) and end of their visit (out; n= 175, 166 and 170, respectively). Correct answers are shown in blue, wrong ones in pink. Tukey whisker plots show upper and lower quartiles as boxes above and below the median line, and whiskers representing the 1.5 interquartile range. Outliers are depicted as individual data points, the mean by the plus symbol. Statistical significance was assessed using RM one-way ANOVA with Greenhouse-Geisser correction and Holm-Sidak's test for multiple comparisons: *, $p<0.05$; **, $p<0.01$; ***, $p<0.001$; ****, $p<0.0001$.

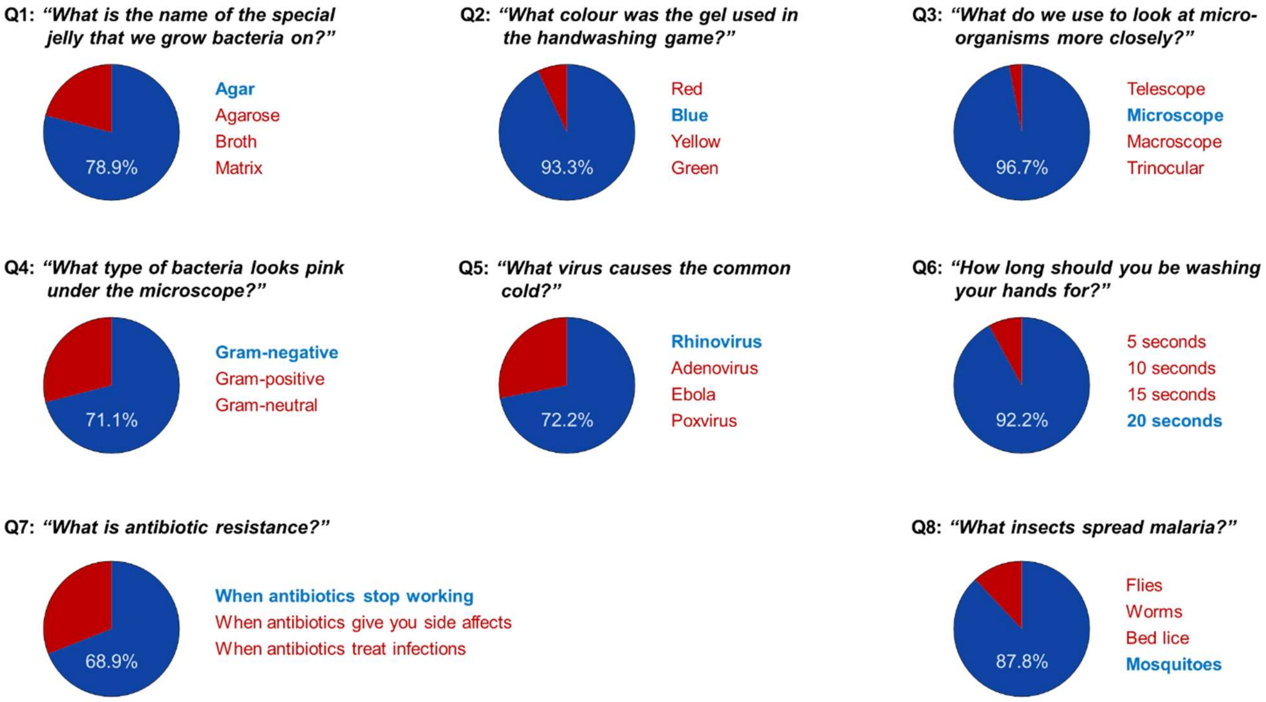

Supplementary Figure 15. Results from fun quizzes conducted at the end of each school visit. Percentages indicate correct answers (blue), wrong answers are highlighted in red. Data are summarised from a total of 90 competing teams consisting of 2–4 players each. No school-specific breakdown is available for these data.

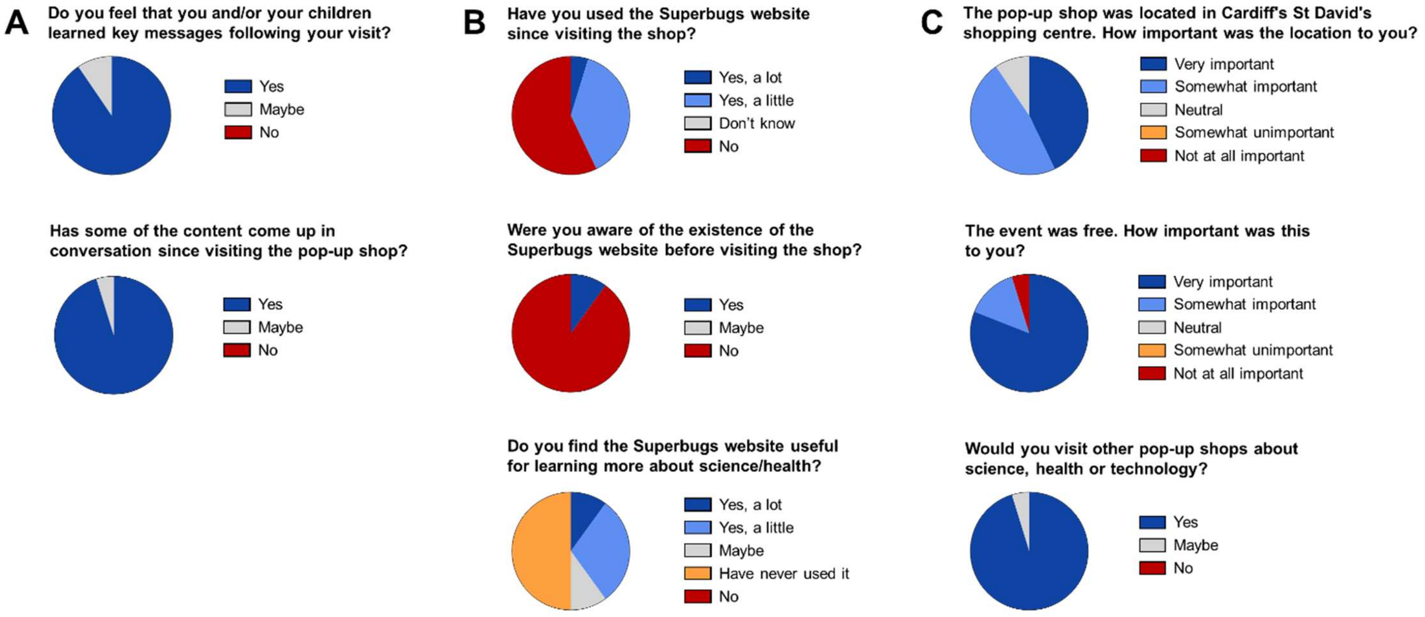

Supplementary Figure 16. Long-term impact of the event. (***A***) Key messages and impact on conversations; (***B***) awareness and perception of the Superbugs educational online resources; (***C***) importance of location, free access and content for the overall approach of taking science to the city. Information collated from 21 visitors providing feedback regarding their views 4.5 months after having visited the pop-up shop.

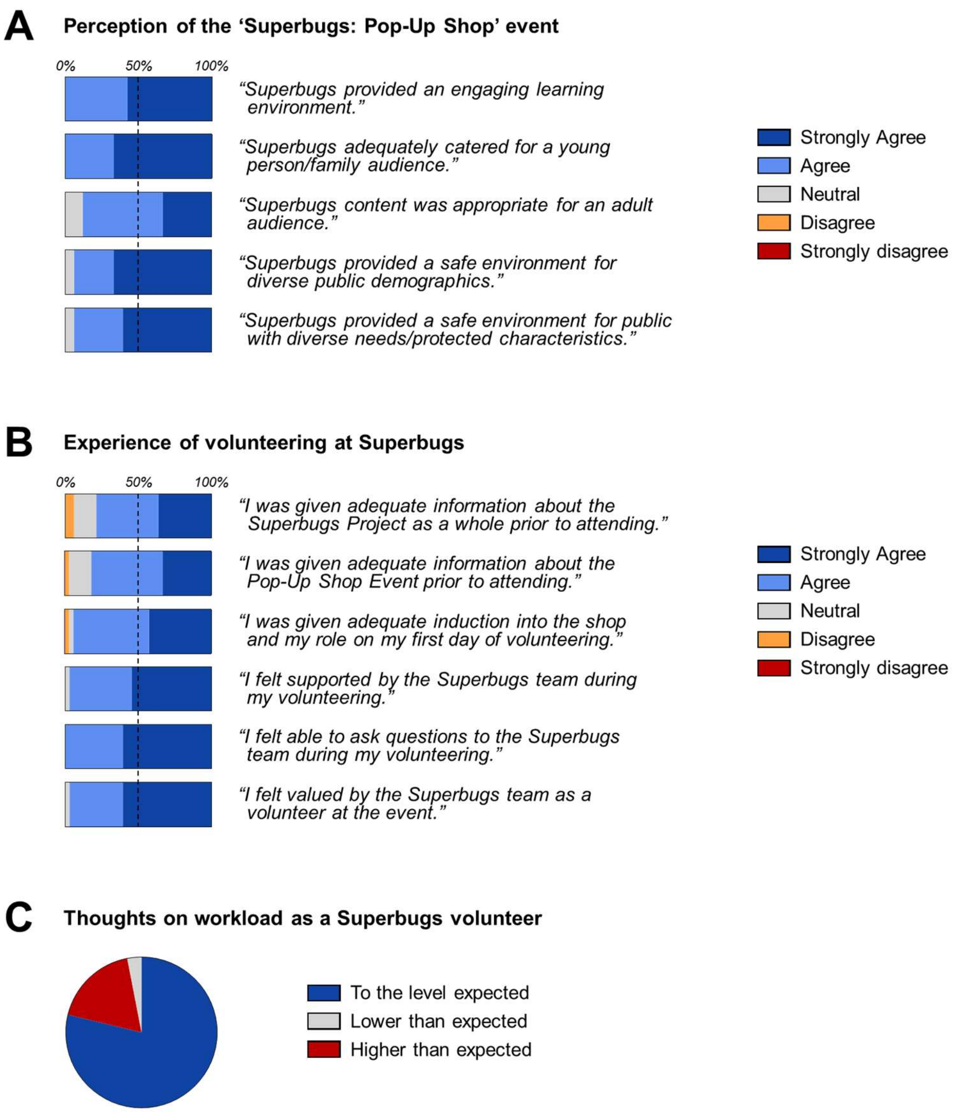

Supplementary Figure 17. Quantitative feedback from helpers in the pop-up shop. (***A***) General perception; (***B***) experience of volunteering; (***C***) perceived workload during the event. Information collated from a total of 33 completed post-event questionnaires.

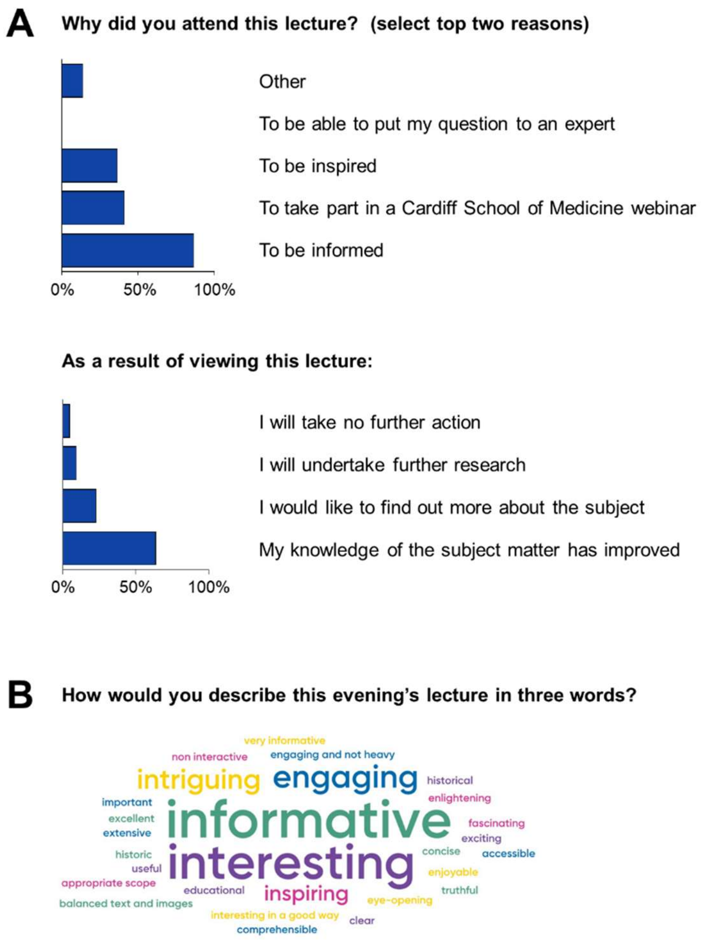

Supplementary Figure 18. Feedback by attendees of the public lecture 'A Microbial Horror Show – Devastating outbreaks of disease that shaped how we fight infections'. This webinar was given by Superbugs co-lead Prof Matthias Eberl on 27 Nov 2025 within the Science in Health public lecture series organised by Cardiff University's School of Medicine, and was attended by 97 people. (***A***) Post-event feedback received from 22 attendants (multiple choice); (***B***) word cloud visualising the respondents' answers.
